# Supplementary material for: A discrete organoplatinum(II) metallacage as a multimodality theranostic platform for cancer photochemotherapy
Source: Nat Commun. 2018 Oct 18;9:4335. doi: 10.1038/s41467-018-06574-7 (PMC6194061; doi:10.1038/s41467-018-06574-7)
Supplement: Supplementary file 1 — Supplementary Information [file 41467_2018_6574_MOESM1_ESM.doc]

**Supplementary Material for**

**A discrete organoplatinum(II) metallacage as a multimodality theranostic platform for cancer photochemotherapy**

Guocan Yu^1^, Shan Yu^2^, Manik Lal Saha^3^, Jiong Zhou^4,5^, Timothy R. Cook^5^, Bryant C. Yung^1^, Jin Chen^2^, Zhengwei Mao^2^, Fuwu Zhang^1^, Zijian Zhou^1^, Yijing Liu^1^, Li Shao^4^, Sheng Wang^1^, Changyou Gao^2^, Feihe Huang^4^, Peter J. Stang^3^, and Xiaoyuan Chen^1^

*^1^ Laboratory of Molecular Imaging and Nanomedicine, National Institute of Biomedical Imaging and Bioengineering, National Institutes of Health, Bethesda, Maryland 20892, United States. Email:* [*shawn.chen@nih.gov*](mailto:shawn.chen@nih.gov)

*^2^ MOE Key Laboratory of Macromolecular Synthesis and Functionalization, Department of Polymer Science and Engineering, Zhejiang University, Hangzhou 310027, P. R. China. Email:* [*zwmao@zju.edu.cn*](mailto:zwmao@zju.edu.cn)

*^3^ Department of Chemistry, University of Utah, 315 South 1400 East, Room 2020, Salt Lake City, Utah 84112, United States. Email:* [*stang@chem.utah.edu*](mailto:stang@chem.utah.edu)

*^4^ State Key Laboratory of Chemical Engineering, Center for Chemistry of High-Performance & Novel Materials, Department of Chemistry, Zhejiang University, Hangzhou 310027, P. R. China. Email:* [*fhuang@zju.edu.cn*](mailto:fhuang@zju.edu.cn)

*^5^ Department of Chemistry, University at Buffalo, State University of New York, 359 Natural Sciences Complex, Buffalo, New York 14260, United States*

**Supporting Information**

| 1. | *Supplementary Methods* | S2 |
| --- | --- | --- |
| 2. | *Syntheses of* ***mPEG****-b-****PEBP*** *and* ***RGD-PEG****-b-****PEBP*** | S8 |
| 3. | *Fabrication of* ***MNPs*** | S17 |
| 4. | *In vitro photochemotherapeutic investigations* | S23 |
| 5. | *In vivo theranostic studies* | S26 |
| 6. | *References* | S50 |

*Supplementary Methods*

5,10,15,20-Tetra(4-pyridyl)-21H,23H-porphine (**TPP**), 2-(bromomethyl)naphthalene (**NPB**), 2,2-dimethoxy-2-phenylacetophenone (DMPA), 2-chloro-2-oxo-1,3,2-dioxaphospholane, disodium terephthalate, 1,3-diphenylisobenzofuran (DPBF), 3-(4,5-dimethylthiazol-2-yl)-2,5-diphenyltetrazolium bromide (MTT), and 2′,7′-dichlorofluorescein-diacetate (DCFH-DA) were purchased from Sigma-Aldrich (St. Louis, MO, USA). *Cis*-[Pt(PEt_3_)_2_(OTf)_2_] (***c*Pt**)^S1^ and 2-ethylbutoxy phospholane (**EBP**)^S2^ were synthesized according to a reported method. Solvents were either employed as purchased or dried according to procedures described in the literature. ^1^H NMR and ^31^P NMR spectra were recorded on a Bruker Avance DMX-500 spectrometer or on a Bruker Avance Ⅲ-400 spectrometry. ^31^P{^1^H} NMR chemical shifts are referenced to an external unlocked sample of 85% H_3_PO_4_. UV-vis-NIR spectra were taken on a Shimadzu UV-3150 spectrophotometer. The fluorescence titration experiments were conducted on a RF-5301 spectrofluorophotometer (Shimadzu Corporation, Japan). High-resolution mass spectrometry experiments were performed with a Bruker 7-Tesla FT-ICR mass spectrometer equipped with an electrospray source (Billerica, MA, USA). Transmission electron microscopy (TEM) investigations were conducted on the FEI Tecnai12 transmission electron microscope (FEI, Hillsboro, Oregon) operating at an accelerating voltage of 120 kV. Images were acquired using a Gatan 2k × 2k cooled CCD camera (Gatan, Pleasanton, CA). Dynamic light scattering (DLS) measurements were carried out using a SZ-100 nano particle analyzer (HORIBA Scientific, Tokyo, Japan). The particle zeta potential values were determined by a Delsa Nano C particle analyzer equipped with a 30 mW dual laser diode. Samples for AFM were prepared by casting 3-10 µL of sample aqueous solution on freshly peeled mica substrate and rinsing, drying and dehumidifying. AFM imaging was conducted in air, using gentle tapping-mode AFM with a PicoForce Multimode AFM (Bruker, CA) consisting of a Nanoscope® V controller, a type E scanner head, and a sharpened TESP-SS (Bruker, CA) or similar AFM cantilever. AFM images were then analyzed by Nanoscope softwares (ver. 7.3-8.15, Bruker, CA). Molecular weights and distributions were determined by gel permeation chromatography (GPC) with a Waters 1515 pump and Waters 1515 differential refractive index detector (set at 30 °C).

**Fabrication of the metallacage.** ***c*Pt** (29.2 mg, 40.0 mM), **TPP** (6.18 mg, 10.0 mM) and **DSTP** (4.20 mg, 20.0 mM) were placed in a 2-dram vial, followed by addition of acetone-*d*_6_ (1.6 mL) and D_2_O (0.4 mL). After stirring at 70 °C for 5 h, the solvent was removed by N_2_ flow. Acetone-*d*_6_ (0.6 mL) was added, and the solution was stiired at 70 °C for an additional 5 h. The formed metallacage (**M**) was precipitated using diethyl ether and the precipitation procedure was repeated four times to purify **M** (yield, 91.6%). Notably, the synthesis of **M** could be easily scaled up, which was favorable for future translational studies. Then **M** was dissolved in CD_3_CN for characterization. For the preparation of metal-chelated matallcages, metal-chelated **TPP** was synthesized first. Briefly, one equivalent molar of CuCl_2_ (or MnCl_2_) was added into the DMF solution containing **TPP**. The solution was heated at 85 ^o^C for 12 h in a nitrogen environment and then was added dropwise into water. Dark purple precipitate was filtered and washed with diethyl ether thoroughly, then purified on a silica gel column using MeOH/CH_2_Cl_2_ (1:2) as eluent to give metal-chelated **TPP** as dark purple solid. The heteroligation-directed self-assembly of the metal-chelated matallcages was similar to that of **M**. Due to its radioactivity, the preparation of ^64^Cu@**M** was conducted by one step. ^64^CuOTf_2_ (0.50 mCi) was added into acetonitrile containing excessive **M** (10.0 mM) and the solution stirred for 2 h to afford ^64^Cu@**M** directly.

It should be noted that partial dissociation of **M** in the **MNPs** could not be completely avoided during circulation in bloodstream due to the existence of competitive ions. Actually, we monitored that around 12% of the loaded **M** was released from the **MNPs** in the physiological environment containing 10% FBS within 24 h, which was a common phenomenon for nanomedicines. However, we firmly trusted that most of the metallacages in the hydrophobic core of **MNPs** could be effectively protected by the amphiphilic copolymers during drug delivery process, because the hydrophobic environment in the core of the NPs can effectively inhibit the penetration of ions into the NPs. We also synthesized a **TPP**-***c*Pt** conjugate as a control during the experiments to test the anticancer efficacy of the resultant **TPP**-***c*Pt**-loaded NPs against U87MG cells. We found that IC_50_ value of **TPP**-***c*Pt**-loaded NPs (0.93 ± 0.16 μM) was much higher than that of **MNPs** (21.4 ± 2.7 nM, based on the molar amount of **MNPs**), emphasizing that the metallacage structure played a significant role in PDT and chemotherapy.

**Preparation of nanoparticles (NPs) and metallacage-loaded nanoparticles (MNPs).** The nanoparticles (NPs) were fabricated through a matrix-encapsulation method. **mPEG**-*b*-**PEBP** (25.0 mg) and **RGD-PEG**-*b*-**PEBP** (5.00 mg) were solublized in 5 mL of acetone, then the solution was injected into MilliQ water (20 mL). After sonication for 5 min, the organic solvent was completely removed *in vacuo*. After sonication for another 5 min, a well dispersed nanoparticle suspension was obtained.

The method for the fabrication of the metallacage-loaded nanoparticles without cRGDfK groups was similar to the preparation of **MNPs** by only using **mPEG**-*b*-**PEBP** as the amphiphilic block copolymer. The method for the fabrication of Cu **MNPs** (^64^Cu **MNPs** or Mn **MNPs**) was similar to the preparation of **MNPs** by using metal-chelated **M** as building block. The radiolabeling efficiency was measured by instant thin-layer chromatography (ITLC) plates using citric acid (0.1 M) as an eluent. Free ^64^Cu elutes to the solvent front (*r*_f_ = 0.6−0.8) and ^64^Cu **MNPs** stayed at the origin (*r*_f_ = 0−0.1).

**Preparation of TPPNPs.** The method for the fabrication of **TPPNPs** was similar to the preparation of **MNPs**. Briefly, **mPEG**-*b*-**PEBP** (25.0 mg), **RGD-PEG**-*b*-**PEBP** (5.00 mg), and pyridinium, 4,​4',​4'',​4'''-​(21H,​23H-​porphine-​5,​10,​15,​20-​tetrayl)​tetrakis[1-​(2-​naphthalenylmethyl)​-​, hexafluorophosphate (**TPPN**, 10.0 mg) were solublized in 5 mL of acetone. The solution was injected into distilled water (20 mL), and the mixture was sonicated for 5 min. The organic solvent was completely removed *in vacuo*. After sonication for another 5 min, **TPPNPs** were obtained as a well dispersed nanoparticle suspension. Unloaded **TPPN** in the solution was eleminated by passing through a PD-10 column. DLS and TEM were used to characterize these **TPPNPs**. UV-vis spectroscopy was utilized to determine the actual loading amount of **TPPN**. Notably, the loading content of **TPPN** could be easily controlled by altering the ratio between the copolymers and **TPPN**.

**Measurement singlet oxygen quantum yield (^1^O_2_ QY).** The ^1^O_2_ QY of the **MNPs** was calculated by using *meso*-tetrakis(*p*-sulfonatophenyl) porphyrin tetrasodium salt (TPPS) as a standard. Upon laser irradiation (671 nm, 0.5 W cm^–2^) for different time, the absorption of the solution was detected using UV-vis spectroscopy. The slope of the absorbance at 414 nm versus irradiation time was determined. The ^1^O_2_ QY was calculated according to equation list as follows:^S3^

*Φ*_Δ_ = *Φ*_(TPPS)_ × *k* _(MNPs)_ × *F*_(TPPS)_/*k* _(TPPS)_ ×*F*_(MNPs)_

where *k* was the slope of the absorbance at 414 nm versus irradiation time, *F* was the absorption correction factor calculated by *F* = 1 – 10^OD^ (OD at the irradiation wavelength).

***In vitro* cell accumulation of the** **MNPs determined by flow cytometry.** Quantitative determination of the **MNPs** amount internalized by the cells was measured using flow cytometry. U87MG cells were seeded in 12-well cell culture plates at a density of 3.00 × 10^5^ cells/well. The cells grew in MEM medium containing FBS (10%) at 37 °C for 24 h in 5% CO_2_ atmosphere. **MNPs** (or the NPs without cRGDfK ligands, the concentration of **M** was kept at 1.00 *μ*M) were added into the wells, then the cells were cultured for different time (1 h, 2 h, 3 h, and 4 h, respectively). Following incubation, cells were washed twice by PBS to eliminate residual **MNPs** (or the NPs without cRGDfK ligands). Cells were harvested by trypsinization and resuspended in PBS (500 μL) for flow cytometry study. Cells without any treatment were utilized as a control. Data was analyzed by using the FlowJo software. To confirm internalization *via* receptor-mediated endocytosis, a competition assay was performed by pre-treating the cells with free cRGDfK (20 µM) for 30 min. The culture media were refreshed and the cells were cultured with **MNPs** (or the NPs without cRGDfK) for another 4 h for flow cytometry analysis.

***In vitro* cell accumulation of the MNPs determined** **by confocal laser scanning microscopy (CLSM).** U87MG cells were treated with the **MNPs** (the concentration of **M** was kept at 1 *μ*M) at 37 °C for 2 h and 4 h. Then the cells were rinsed with PBS (three times) and fixed with formaldehyde (4.0%) for 15 min. The cells were carefully washed by PBS and stained with DAPI (1 μg mL^–1^) and LysoTracketr Green (200 nM) for 20 min, respectively. The images were collected on a LSM-510 confocal laser scanning microscope (Zeiss, Germany). To confirm internalization *via* receptor-mediated endocytosis, a competition assay was performed by pre-treating the cells with cRGDfK (20 µM) for 30 min. The culture media were refreshed after 30 min and treated with the **MNPs** for 4 h and analyzed by CLSM.

**DNA viscosity experiments.** Viscosity measurements were carried out to confirm the non-intercalative nature of **M**.^S4^ ct-DNA (200 µM) was treated with varying concentrations of **M** in PBS at pH = 7.2. The average flow times of each sample (*t*) [corrected by average flow time of buffer alone (*t*_0_) and recorded in triplicate] were calculated using Schott AVS 310 automated viscometer. The relative viscosities (*η*) were obtained from the average flow time values using the equation: *η* = (*t*-*t*_0_)/*t*_0_. The data was presented as a plot of relative specific viscosity [(*η*/*η*_0_)^1/3^] *vs.* [Pt]/[DNA], where *η* and *η*_0_ are the viscosities of ct-DNA in the presence and absence of **M** or the matallacage.

**Analyses of cellular uptake of the platinum content by ICP-MS.** U87MG cells were seeded in 12-well cell culture plates (2.00 × 10^5^ cells/well). The cells grew in MEM media containing FBS (10%) at 37 °C for 24 h. **MNPs** (the concentration of Pt was kept at 10 *μ*M) were added and the cells were further cultured for 1 h, 2 h, 3 h, and 4 h, respectively. Then, the cells were washed, digested and collected, and the Pt content in cells was determined by ICP-MS. To confirm internalization *via* receptor-mediated endocytosis, a competition assay was performed that involved pre-treating with 20 µM free cRGDfK. The culture medium were refreshed after 30 min and treated with the **MNPs** for 4 h, and the Pt content in cells was determined by ICP-MS.

**Determination of the percentage of apoptotic cells at different stages by Annexin-V FITC/PI assay.** U87MG cells were seeded in 6-well cell culture plates (5.00 × 10^5^ cells/well). After 12 h incubation, the medium was replaced by fresh growth media with/without ***c*Pt**, **TPPNPs**, and **MNPs**. The platinum concentration of ***c*Pt** and **MNPs** was kept at 600 nM, and the concentration of **TPPN** in **TPPNPs** was 150 nM. The cells were irradiated with light at 671 nm (0.1 W cm^–2^, 3 min) after 12 h incubation for the groups treated with **TPPNPs** (mono-PDT) and **MNPs** (photochemotherapy). The cells were incubated another 12 h after irradiation and harvested with trypsin (0.25%). Then the cells were carefully washed by PBS and stained by annexin-V FITC and propidium iodide (PI) according to the manufacturer’s protocol. Flow cytometry was performed and data were analyzed using BD FACSDiva. The cells without any treatment were utilized as a control.

**PET/CT imaging.** Following 1 h of radiotracer uptake under continuous anesthesia inhalation, mice were placed prone in the microPET-CT scanner (Siemens Preclinical Solution USA, Inc., Knoxville, TN, USA). MicroCT scans were conducted with an X-ray tube voltage of 80 kV, a current of 500 μA, an exposure time of 150 ms, and 120 rotation steps. A 10 min PET static acquisition was performed and the images were reconstructed using ordered set expectation maximization algorithm for 3D PET reconstruction. Images were analyzed using the Inveon Research Workplace 4.1 (Siemens, Erlangen, Germany).

**Tissue histopathology evaluation.** In the histological assay, the heart, liver, spleen, lung, kidney and tumor tissues were fixed in paraformaldehyde (4%) for 24 h. Then the specimens were dehydrated in ethanol, embedded in paraffin, and cut into thick sections (5 mm). The fixed sections were deparaffinized and hydrated according to a standard protocol and stained with hematoxylin and eosin (H&E) for observation. Apoptosis of the tumor cells in the mice after different treatments was determined by the TUNEL method according to the manufacturer’s instructions. For analysis of cell proliferation, sections were incubated with anti-Ki67 antibody (1:25, ab28364, Abcam, United Kingdom), then incubated with the secondary antibody of a Rb IgG (H+L)/HRP (ZB-2301, ZSGB-BIO, China) according to the manufacturer’s instructions.

**Statistical analysis.** Data are reported as mean ± SD. The differences among groups were determined using one-way ANOVA analysis followed by Tukey' s post-test: **P* < 0.05, ***P* < 0.01, ****P* < 0.001.

*Synthesis of* ***TPPN****,* ***mPEG****-b-****PEBP*** *and* ***RGD-PEG****-b-****PEBP***

**
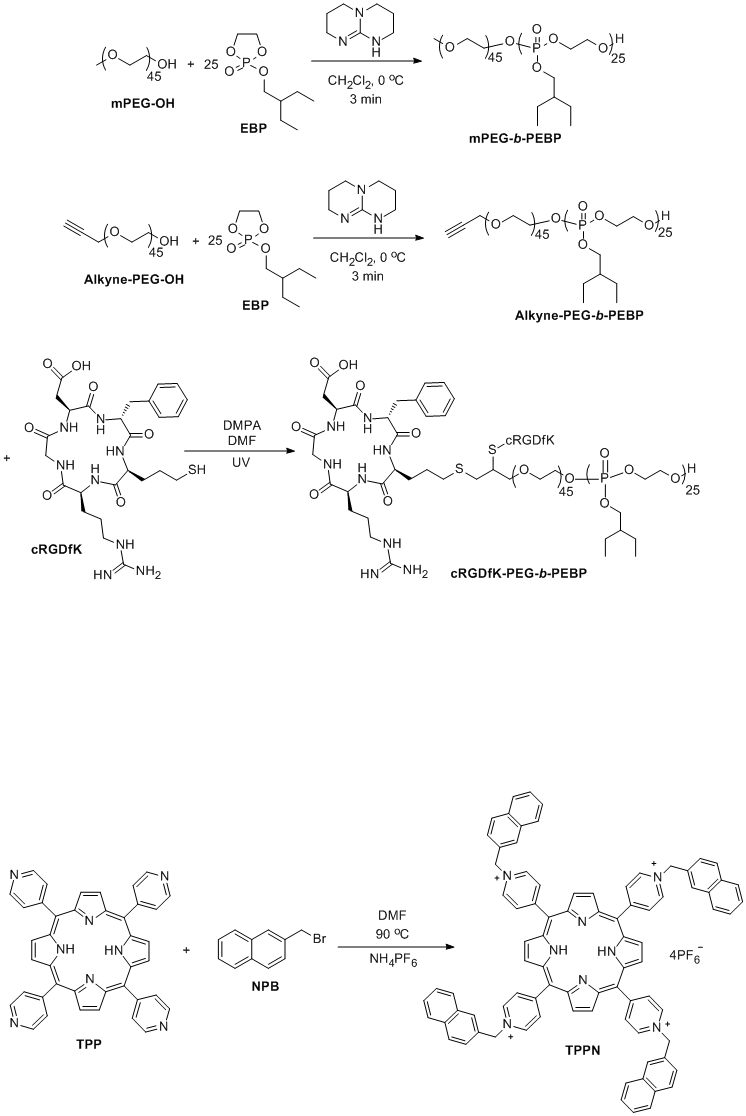
**

**Supplementary Figure 1.** Synthetic route to **TPPN**.

**Synthesis of TPPN:** In order to improve the solubility in organic solvent and increase loading efficiency of the photosensitizer, **TPP** was modified using 2-(bromomethyl)naphthalene, and the counterions were exchanged into hexafluorophosphate. **TPP** (618 mg, 1.00 mmol) and **NPB** (2.19 g, 10 mmol) were mixed in DMF and stirred at 90 ^o^C for 12 hours in a nitrogen environment. The solution was then cooled and added dropwise into acetonitrile. Dark purple precipitate was filtered and washed with diethyl ether. The obtained solid was dissolved in water, excessive NH_4_PF_6_ was added into the aqueous solution, the formed dark purple precipitate was filtered and washed with water to afford **TPPN** as a purple solid (yield, 76%). The proton NMR spectrum of **TPPN** is shown in Supplementary Figure 2. ^1^H NMR (300M, DMSO-*d*_6_, room temperature) *δ* (ppm): 9.75 (d, *J* = 6.0 Hz, 8H), 9.24 (s, 8H), 9.05 (d, *J* = 6.0 Hz, 8H), 8.47 (s, 4H), 8.19 (d, *J* = 6.0 Hz, 4H), 8.11–8.07 (m, 8H), 8.04–8.01 (m, 4H), 7.71–7.66 (m, 8H), 6.42 (s, 8H).


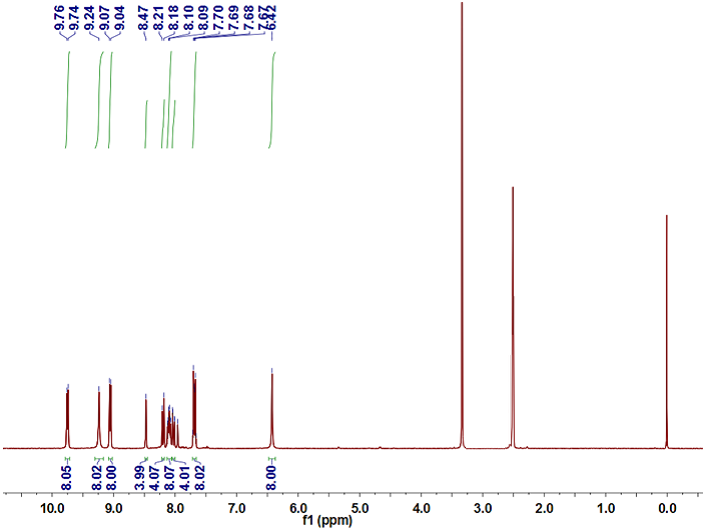


**Supplementary Figure 2.** ^1^H NMR spectrum (300 MHz, DMSO-*d*_6_, room temperature) of **TPPN**.

**
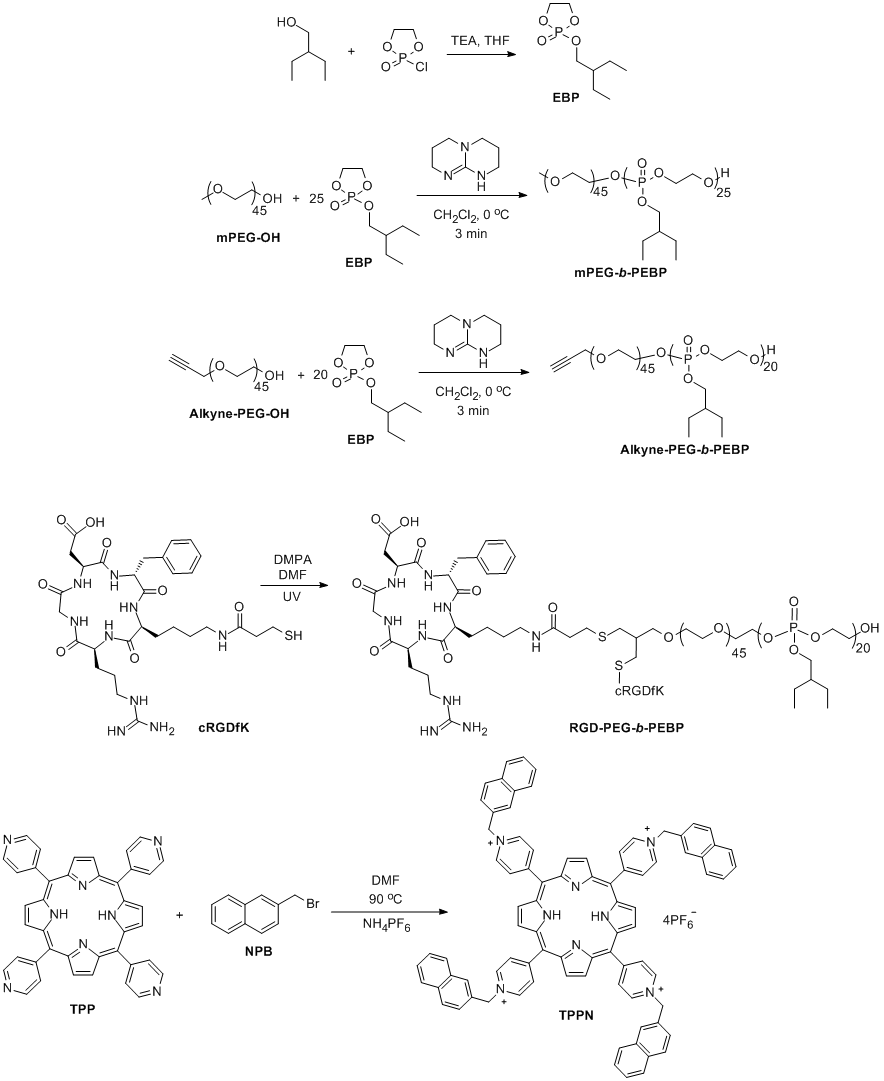
**

**Supplementary Figure** **3.** Synthetic routes to **mPEG**-***b***-**PEBP** and **RGD-PEG**-***b***-**PEBP**.

Synthesis of **EBP**: To a stirred solution of 2-ethyl-1-butanol (7.87 g, 78 mmol) and triethylamine (7.80 g, 78 mmol) in 250 mL of anhydrous THF at 0 °C was added a solution of 2-chloro-2-oxo-1,3,2-dioxaphospholane (10.0 g, 70 mmol) in 50 mL of anhydrous THF dropwise, and the reaction mixture was allowed to stir for 12 h in an ice bath. After complete conversion of 2-chloro-2-oxo-1,3,2-dioxaphospholan as confirmed by TLC, the reaction mixture was filtered and the filtrate was concentrated. The concentrated filtrate was distilled under reduced pressure to obtain a colorless viscous liquid (118–121 °C, 0.4 mm Hg, 9.72 g, Yield: 67 %). The proton NMR spectrum of **EBP** is shown in Supplementary Figure 4a. ^1^H NMR (300M, CDCl_3_, room temperature) *δ* (ppm): 4.42–4.33 (m, 4H), 4.07–4.03 (m, 2H), 1.42–1.46 (m, 1H), 1.36–1.32 (m, 4H), 0.86 (t, *J* = 6 Hz, 6H). The ^31^P NMR spectrum of **EBP** is shown in Supplementary Figure 4b.

**
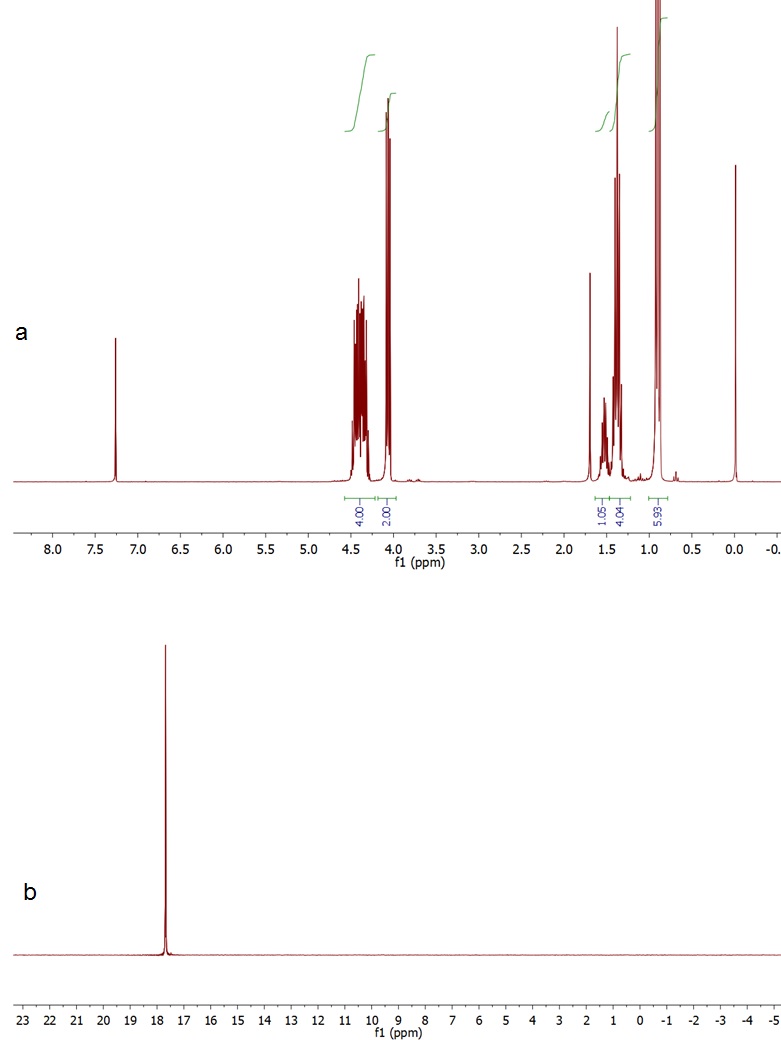
**

**Supplementary Figure 4.** (a) ^1^H NMR spectrum (300 MHz, CDCl_3_, room temperature) of **EBP**. (b) ^31^P NMR spectrum (121.4 MHz, CDCl_3_, room temperature) of **EBP**.

Synthesis of methoxypolyethylene glycol-block-poly(2-ethylbutoxy phospholane) (**mPEG**-***b***-**PEBP**) and alkyne-polyethylene glycol-block-poly(2-ethylbutoxy phospholane) (**Alkyne-PEG-*b*-PEBP**): A solution of **EBP** (521 mg, 2.5 mmol) and **mPEG-OH** (or **Alkyne-PEG-OH**, 200 mg, 0.10 mmol) in 3.0 mL anhydrous dichloromethane was transferred *via* syringe into a flame-dried vial equipped with a stir bar and rubber septum, under N2, in a water/ice bath. A solution of 1,5,7-triazabicyclo[4.4.0]dec-5-ene (TBD) (20.1 mg, 0.15 mmol) in 0.5 mL anhydrous dichloromethane was injected quickly into the vial. After being stirred for 3 min, another 3 mL of anhydrous dichloromethane was injected into the vial to dilute the reaction mixture, and the vial was transferred to a cooling bath composed of equal amounts of ice/water and sodium chloride at −10 °C. The reaction was quenched by adding an excess amount of acetic acid dissolved in DCM after another 3 min, and an aliquot of the mixture was used to determine the conversion of **EBP** by ^31^P NMR. The **mPEG-*b*-PEBP** and **Alkyne-PEG-*b*-PEBP** were purified by precipitation from acetone into a pentane/diethyl ether mixture (3:1 volume ratio) three times and then dried under vacuum. The molecular weight and composition of **mPEG-*b*-PEBP** and **Alkyne-PEG-*b*-PEBP** were determined ^1^H NMR and ^31^P NMR spectroscopies (Supplementary Figure 5−9).


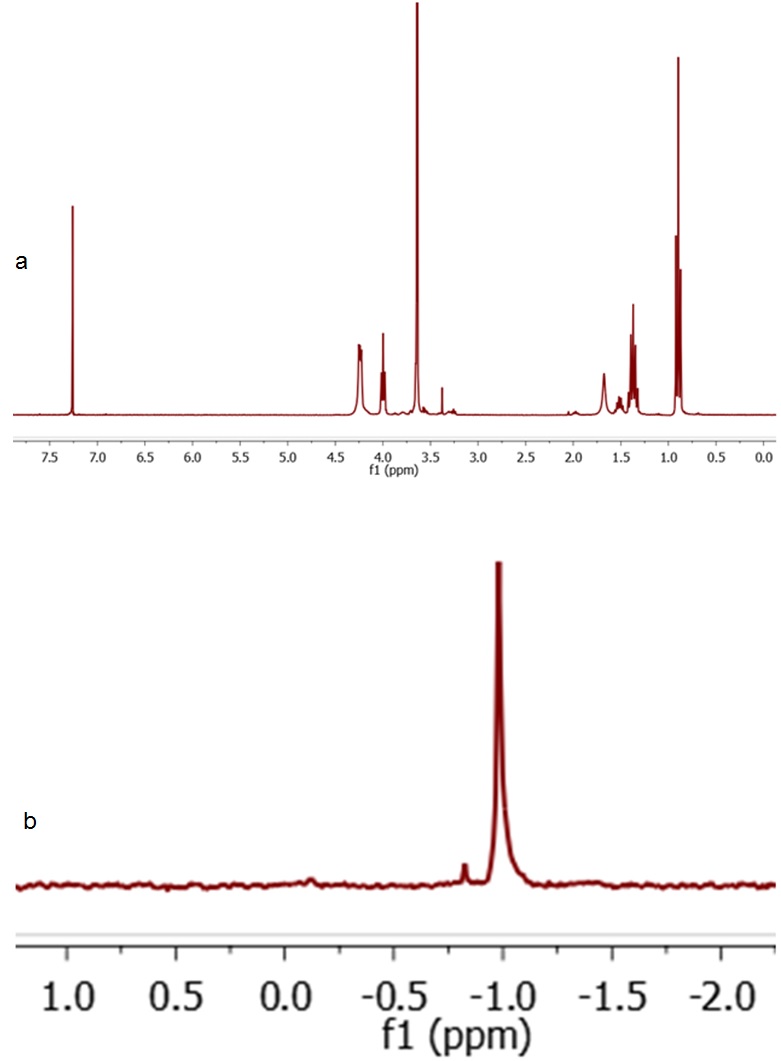


**Supplementary Figure 5.** (a) ^1^H NMR spectrum (300 MHz, CDCl_3_, room temperature) of **mPEG-*b*-PEBP**. (b) ^31^P NMR spectrum (121.4 MHz, CDCl_3_, room temperature) of **mPEG-*b*-PEBP**.


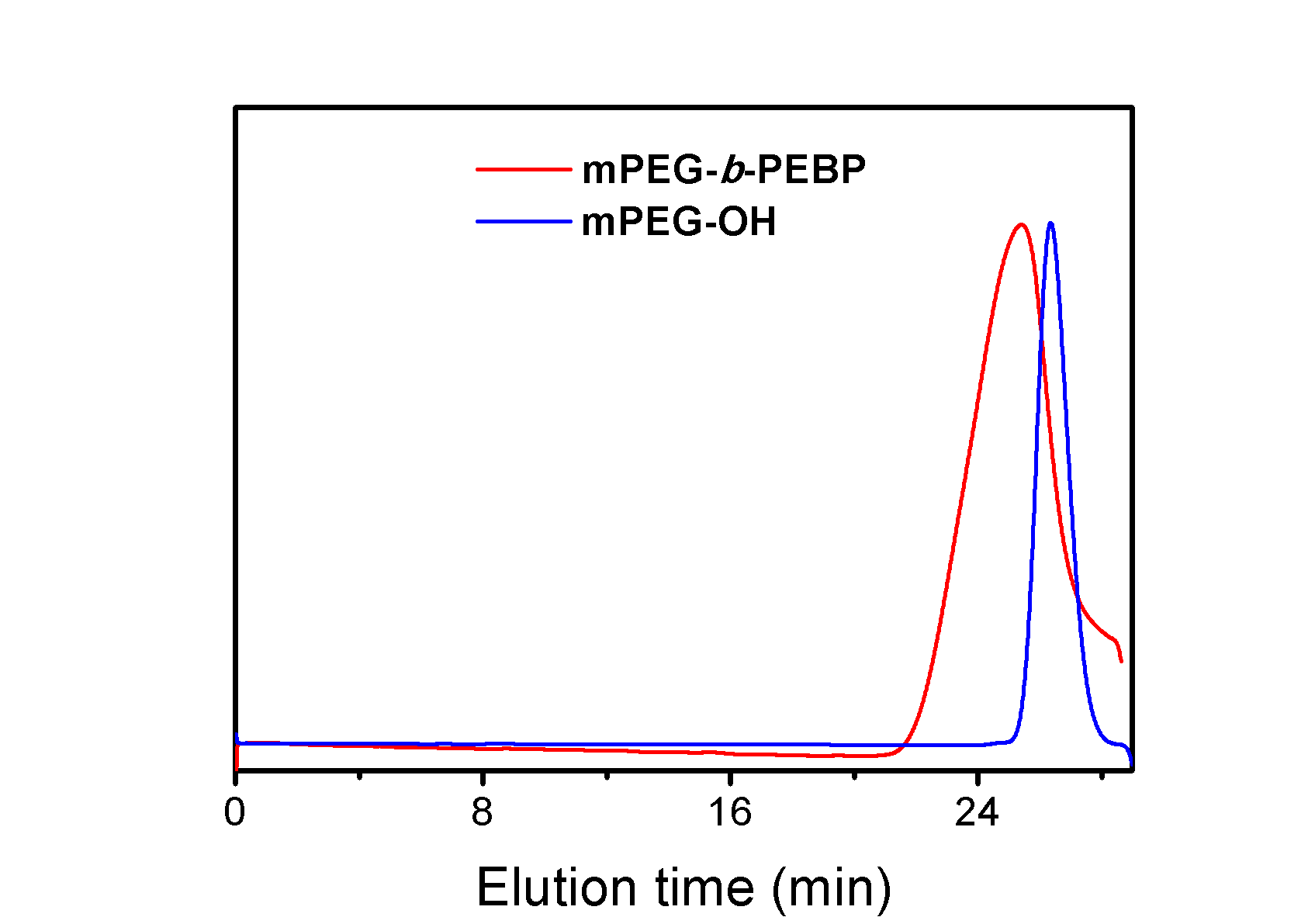


**Supplementary Figure 6.** GPC curves of **mPEG-OH** and **mPEG-*b*-PEBP**.


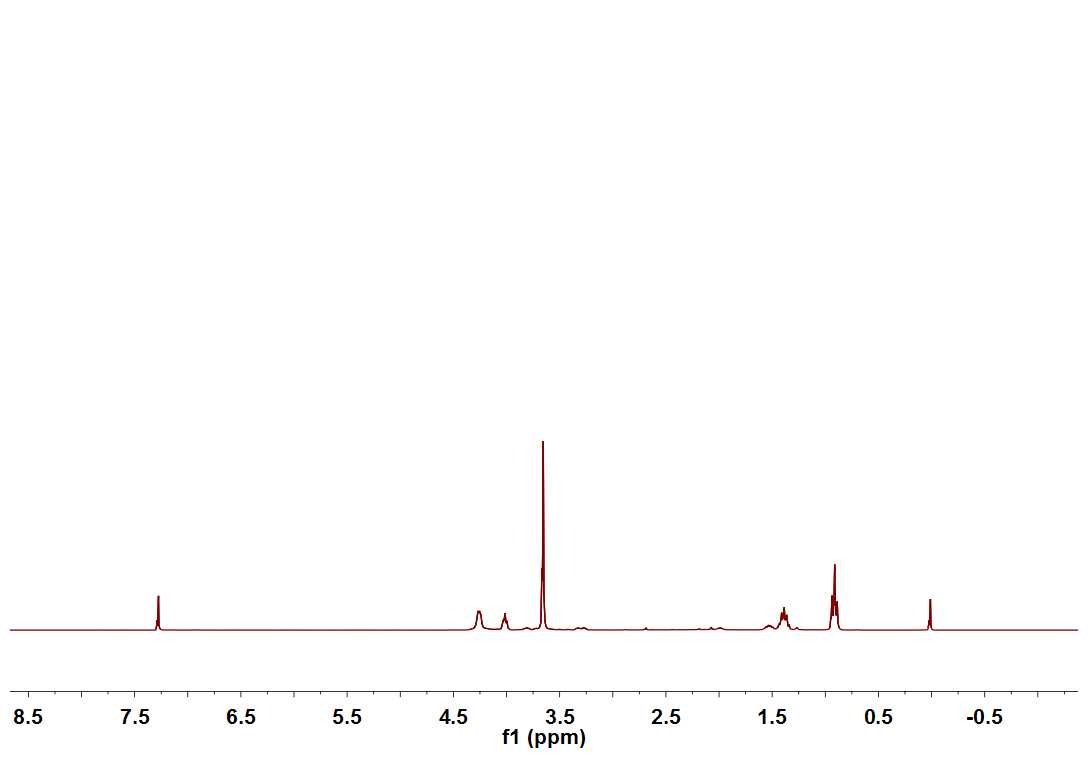


**Supplementary Figure 7.** ^1^H NMR spectrum (300 MHz, CDCl_3_, room temperature) of **Alkyne-PEG-*b*-PEBP**.


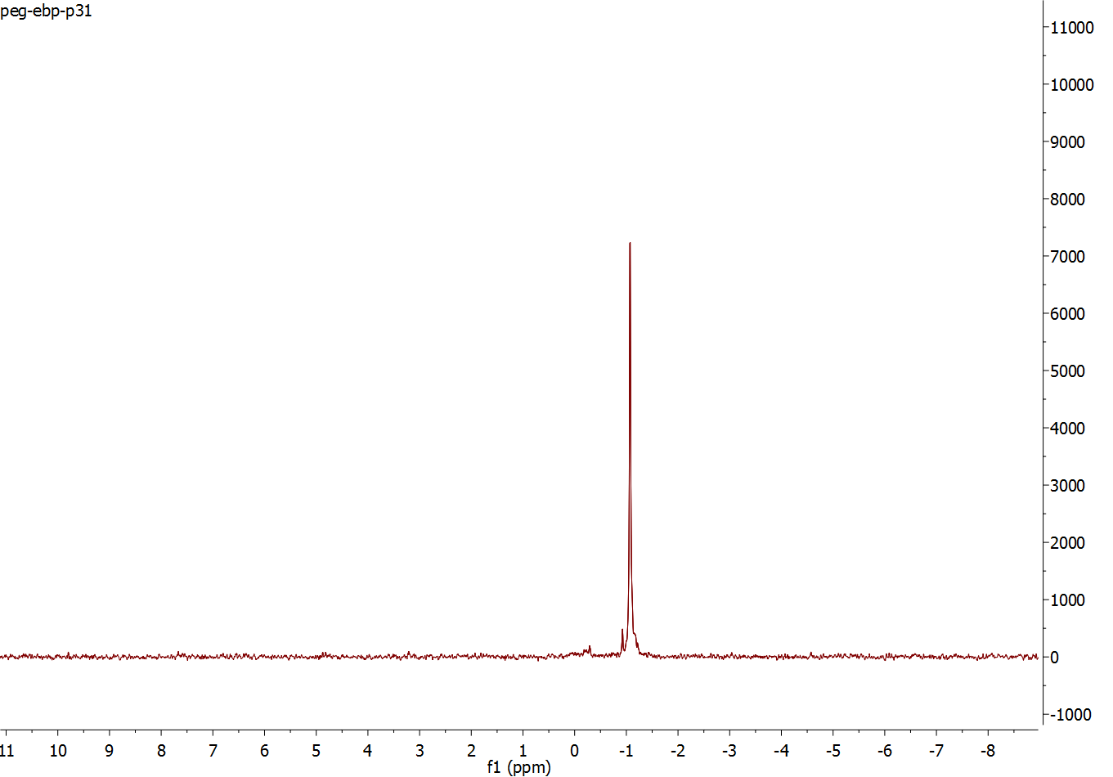


**Supplementary Figure 8.** ^31^P NMR spectrum (100 MHz, chloroform-*d*, room temperature) of **Alkyne-PEG-*b*-PEBP**.


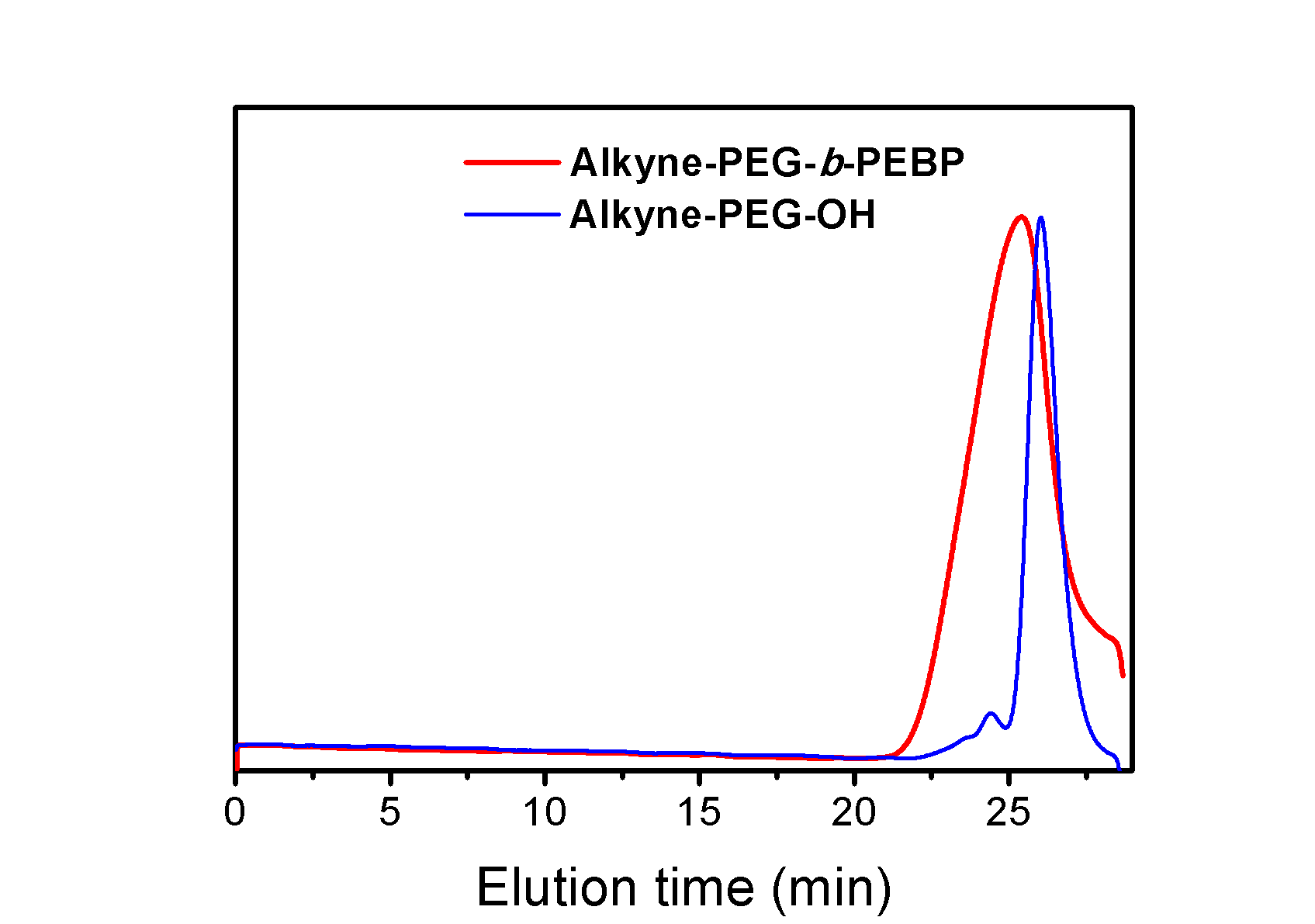


**Supplementary Figure 9.** GPC curves of **Alkyne-PEG-OH** and **Alkyne-PEG-*b*-PEBP**.

**Synthesis of RGD-PEG-*b*-PEBP:** cRGDfK (61.0 mg, 0.10 mmol) and **Alkyne-PEG-*b*-PEBP** (360 mg, 0.05 mmol) were added to a solution of DMPA (0.5 eq., with respect to alkyne residues) in DMF (10 mL). The resulting mixture was placed under an UV lamp and irradiated at 365 nm for 3 h, dialyzed against water for 2 d in presoaked dialysis tubing (MWCO = 3 kDa), and then lyophilized to yield a white powder with a 93% yield. The molecular weight and composition of **RGD-PEG-*b*-PEBP** were determined by GPC and ^1^H NMR studies (Supplementary Figure 10 and 11).


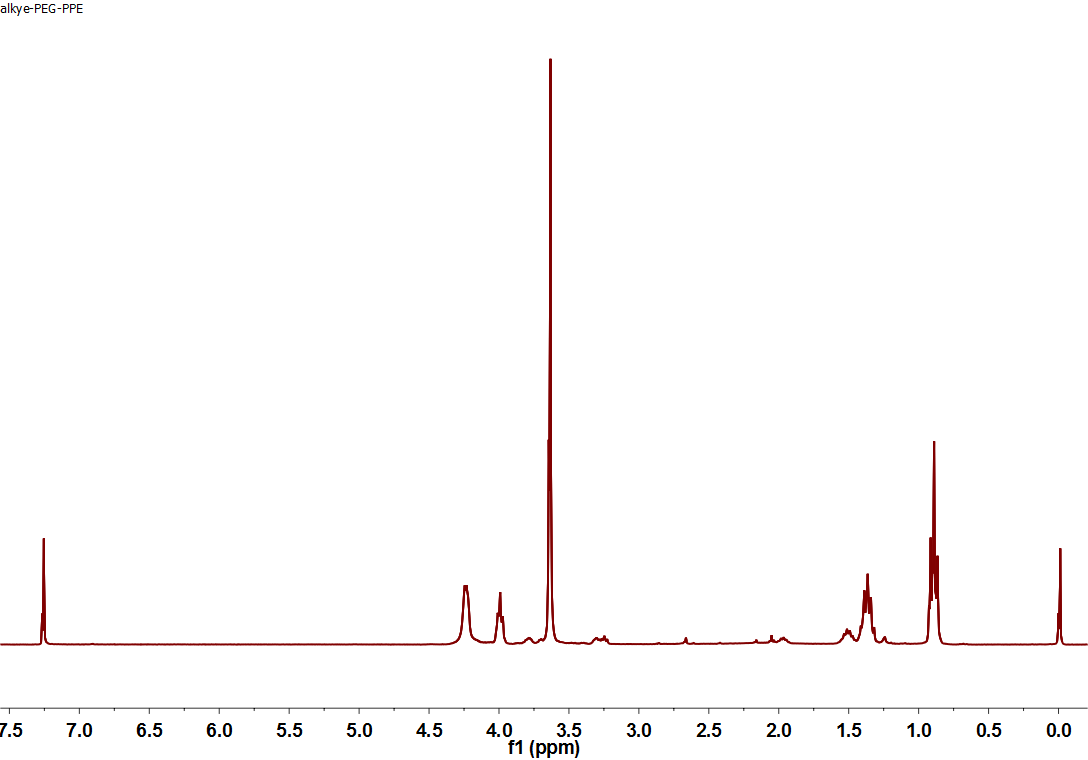


**Supplementary Figure 10.** ^1^H NMR spectrum (300 MHz, chloroform-*d*, room temperature) of **RGD-PEG-*b*-PEBP**.


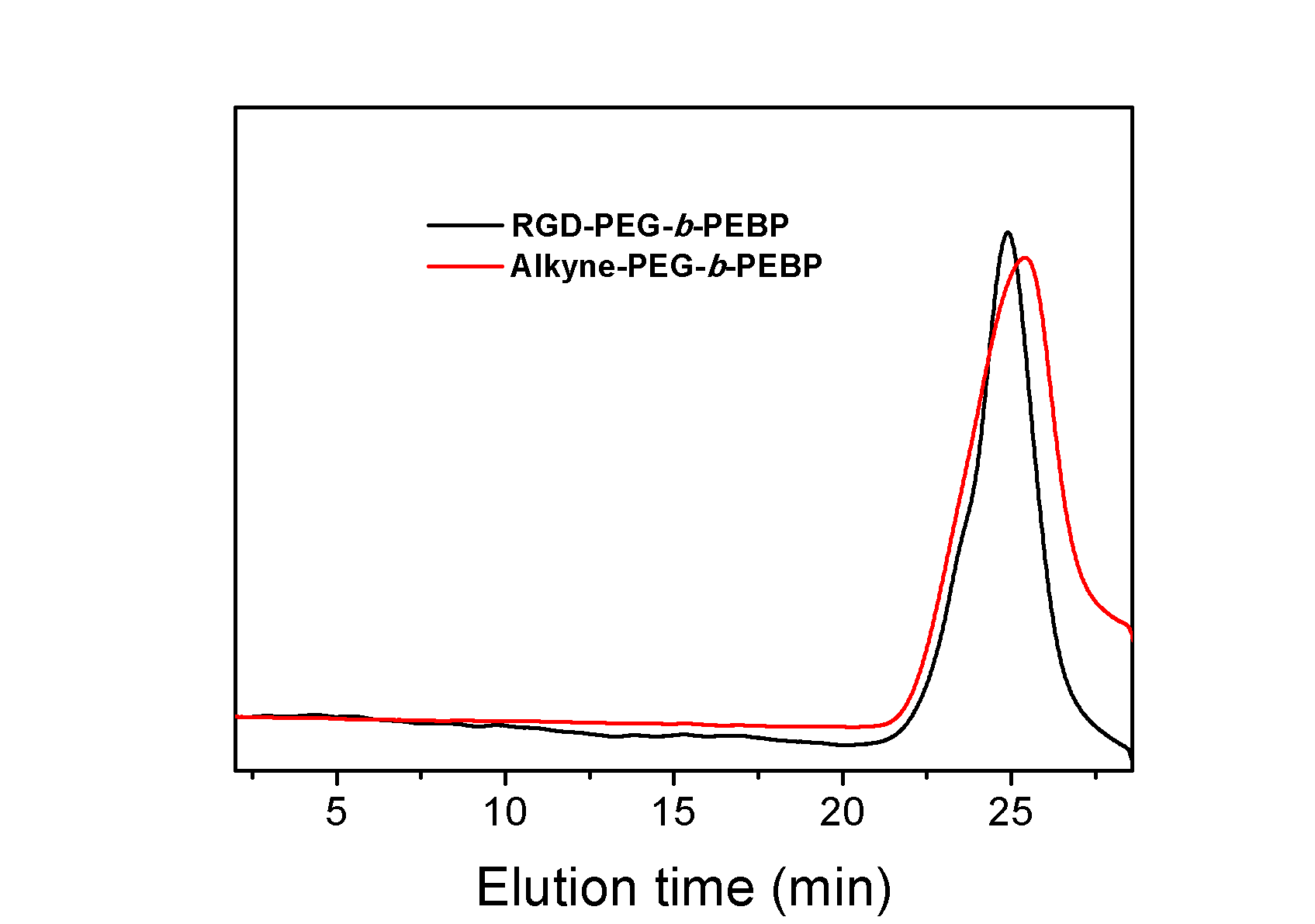


**Supplementary Figure 11.** GPC curves of **Alkyne-PEG-*b*-PEBP** and **RGD-PEG-*b*-PEBP**.

*Fabrication of* ***MNPs***

*
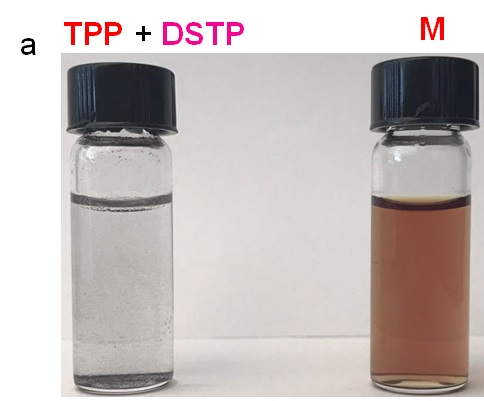
*


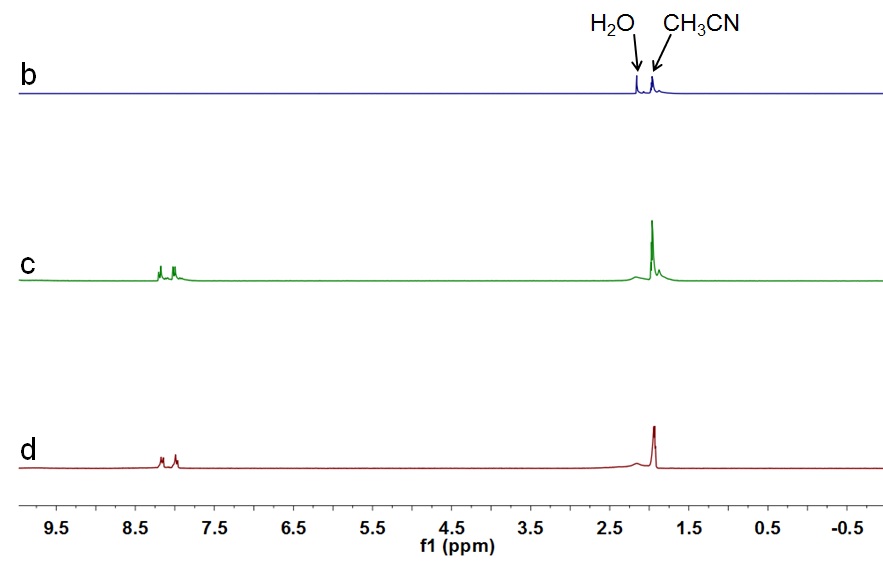


**Supplementary Figure 12.** (a) Photoes of the acetonitrile solution containing **TPP**/**DSTP** mixture and **M**. ^1^H NMR (300 MHz, CD_3_CN, room temperature) spectrum of (b) **TPP**, (c) **TPP** + **DSTP** (2 equiv.), and (d) **DSTP**.


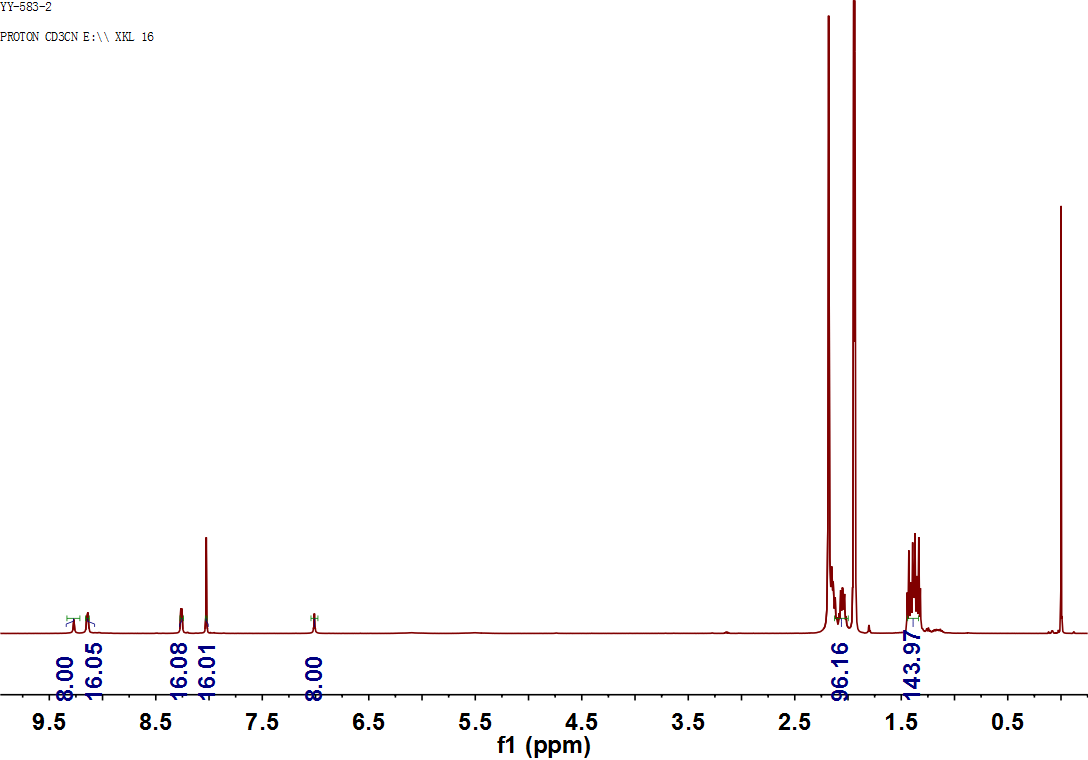


**Supplementary Figure 13.** ^1^H NMR (500 MHz, CD_3_CN, room temperature) spectrum of **M**.

The solubility of **TPP** and **DSTP** was poor in CH_3_CN, especially for **TPP**. The resonance signals related to the protons of **TPP** could not be monitored in ^1^H NMR spectrum (Supplementary Figure 12b−12d). On the contrary, their solubility in CH_3_CN was significantly improved by the formation of metallacage (Supplementary Figure 12a).


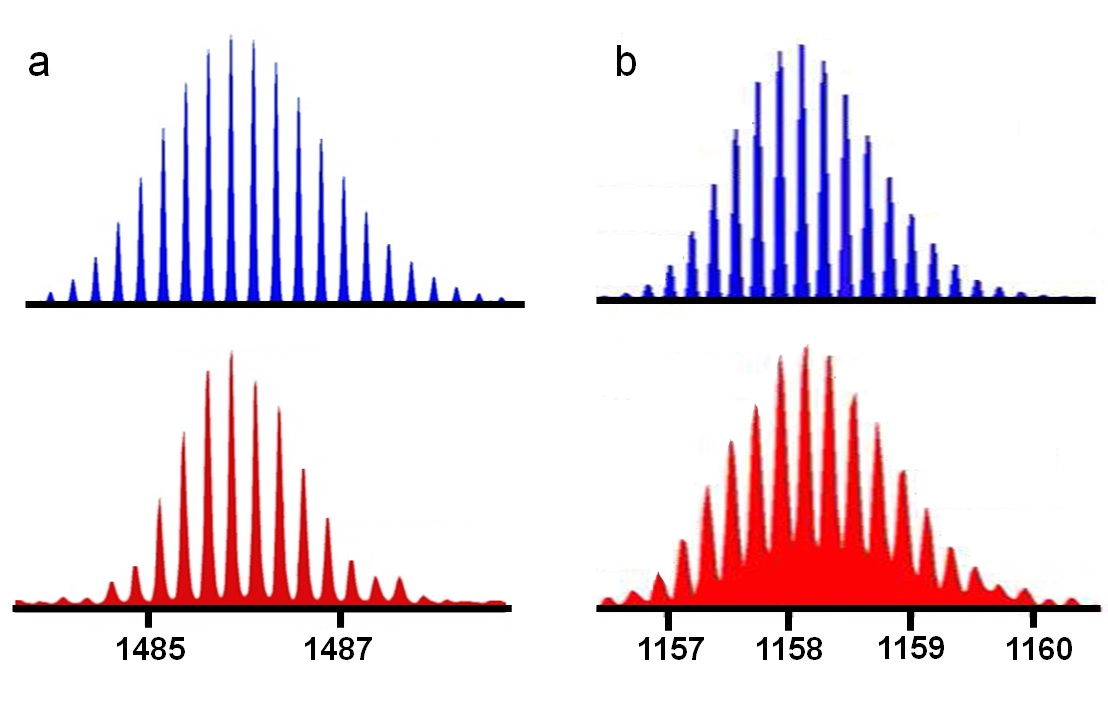


**Supplementary Figure 14.** Experimental (red) and calculated (blue) ESI-TOF-MS spectra of (a) [**M** − 4OTf]^4+^, (b) [**M** − 5OTf]^5+^.


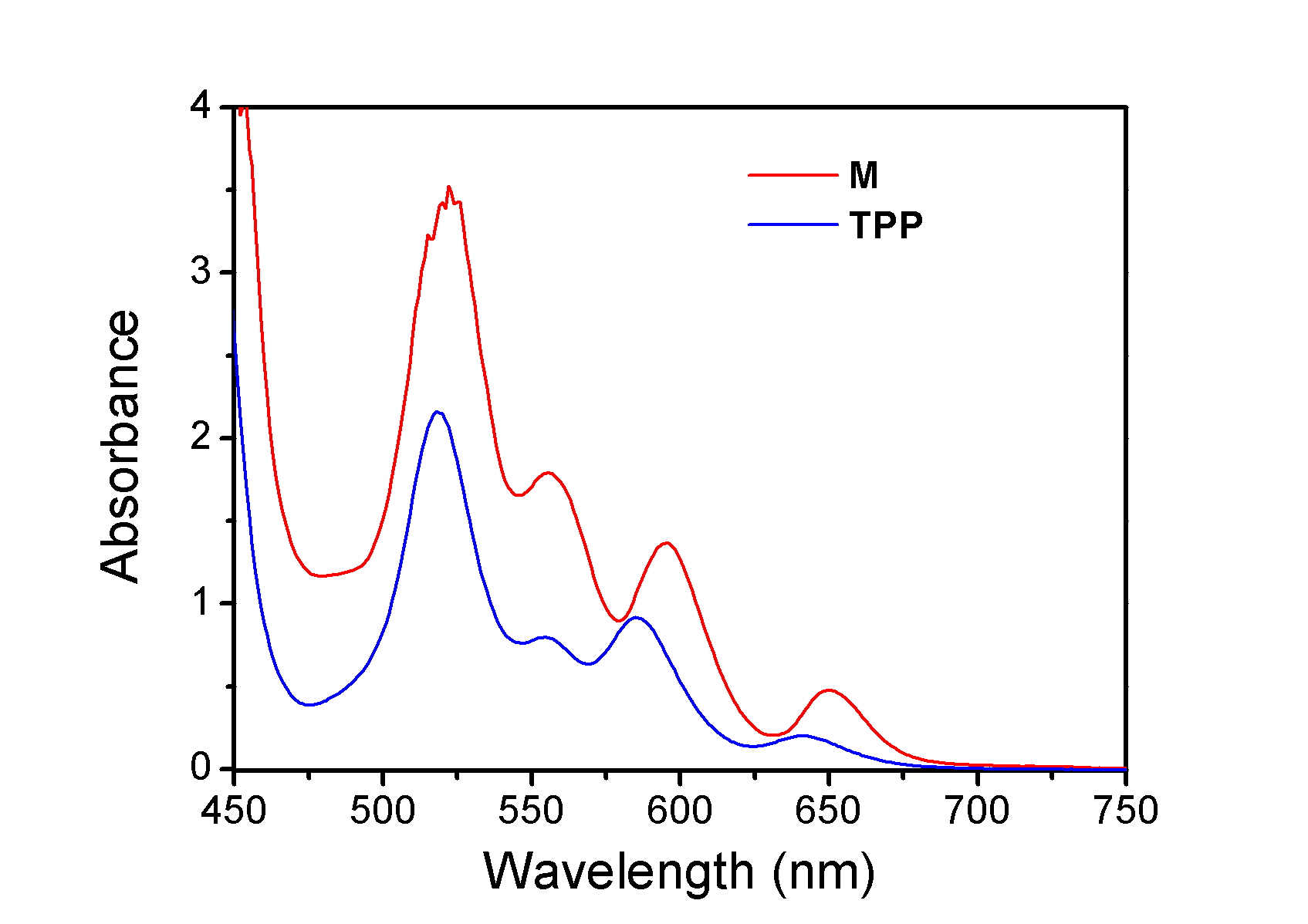


**Supplementary Figure S15.** UV-vis spectra of **M** and **TPP** in the mixture of DCM and methanol (1:1, *v*/*v*)

**Supplementary Table 1. Loading capability and stability evaluations.**

**
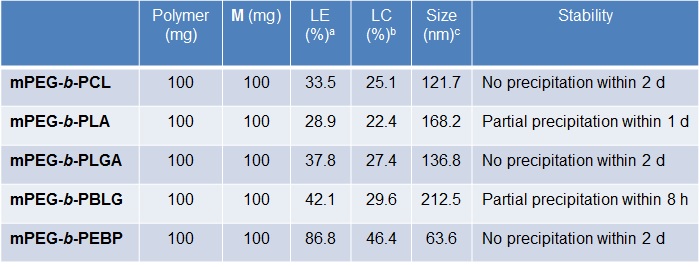
**

^a^ Loading efficient (LE) = *m*_load_/*m*_add_ * 100%, where *m*_add_ and *m*_load_ represent the mass of **M** added and loaded during the preparation of **MNPs**.

^b^ Loading content (LC) = *m*_load_/(*m*_load_ + *m*) * 100%, where *m* represents the mass of the copolymer used during the preparation of **MNPs**.

^c^ The size of **MNPs** was measured by using DLS.

Compared to other classic diblock copolymers, such as poly(ethylene glycol)-*block*-poly(lactic acid) (PEG-*b*-PLA), poly(ethylene glycol)-*block*-poly(caprolactone) (PEG-*b*-PCL), poly(ethylene glycol)-*block*-poly(_D,L_-lactic-coglycolic acid) (PEG-*b*-PLGA), and poly(ethylene glycol)-*block*-poly(*γ*-benzyl-_L_-glutamic acid) (PEG-*b*-PBLG) that have been widely used in the preparation of nanomedicines, these two biodegradable polymers exhibit numerous advantages. Firstly, the electronegative hydrophobic segments play a significant role in the encapsulation of cationic **M**, meaning electrostatic effects assist in stabilization. Secondly, the flexibility of the polyphosphoesters segment is beneficial to encapsulate **M**. The loading content was determined to be 46.4% by UV-vis spectroscopy, which was much higher than the loadings for those with PEG-*b*-PLA, PEG-*b*-PCL, PEG-*b*-PLGA, and PEG-*b*-PBLG at the same conditions (Supplementary Table S1). Thirdly, the polyphosphoesters backbone can effectively avoid non-specific adsorption and uptake, which is favorable for extending the circulation time of the resultant **MNPs**.


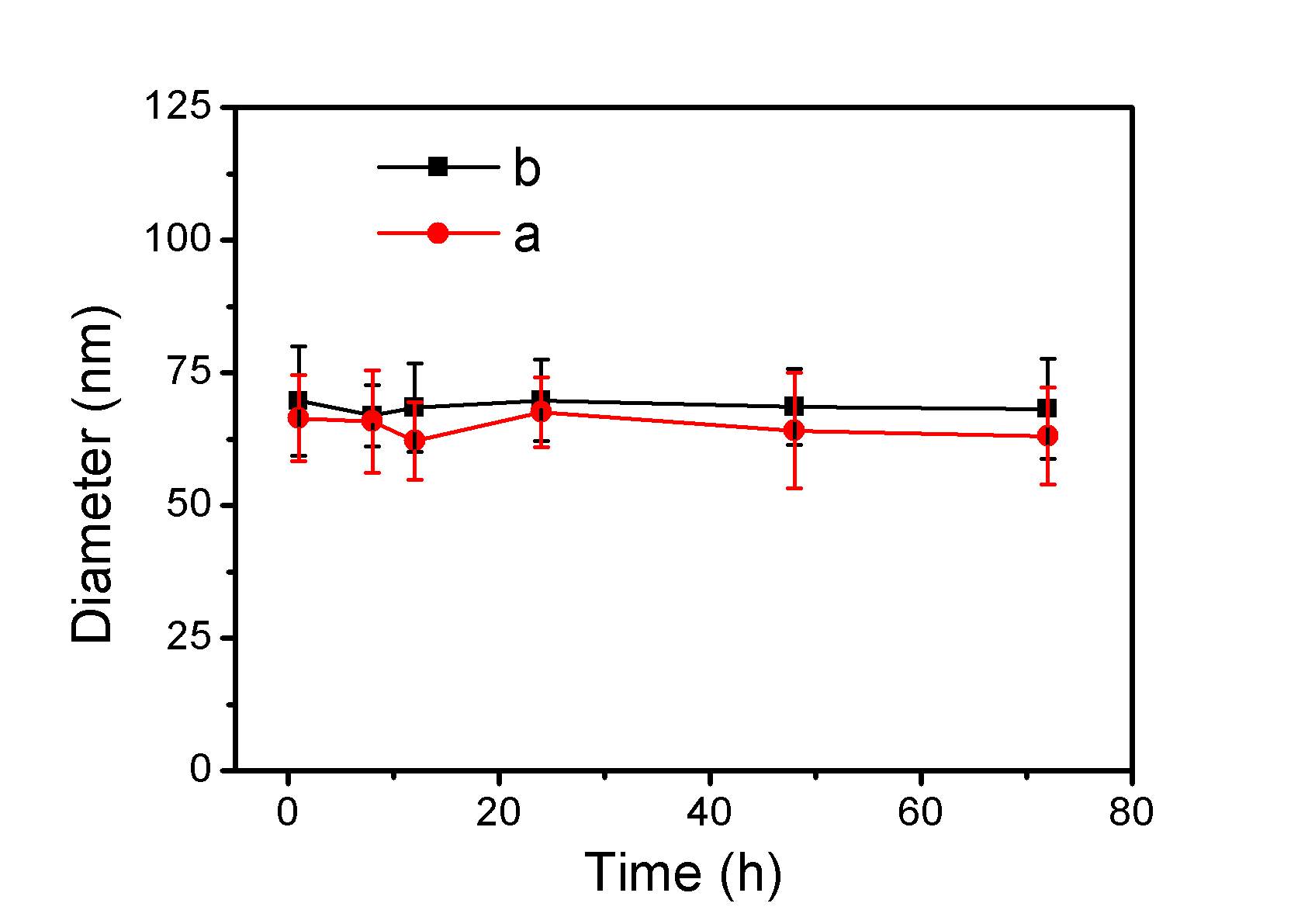


**Supplementary Figure 16.** The average diameters of **MNPs** in PBS containing (a) 10% FBS or (b) 50% FBS after different time periods of incubation.

As shown in Supplementary Figure 16, negligible changes in average diameter of **MNPs** were detected in PBS containing 10% or 50% FBS after different time periods of incubation, demonstrating that **MNPs** were stable in physiological environment.


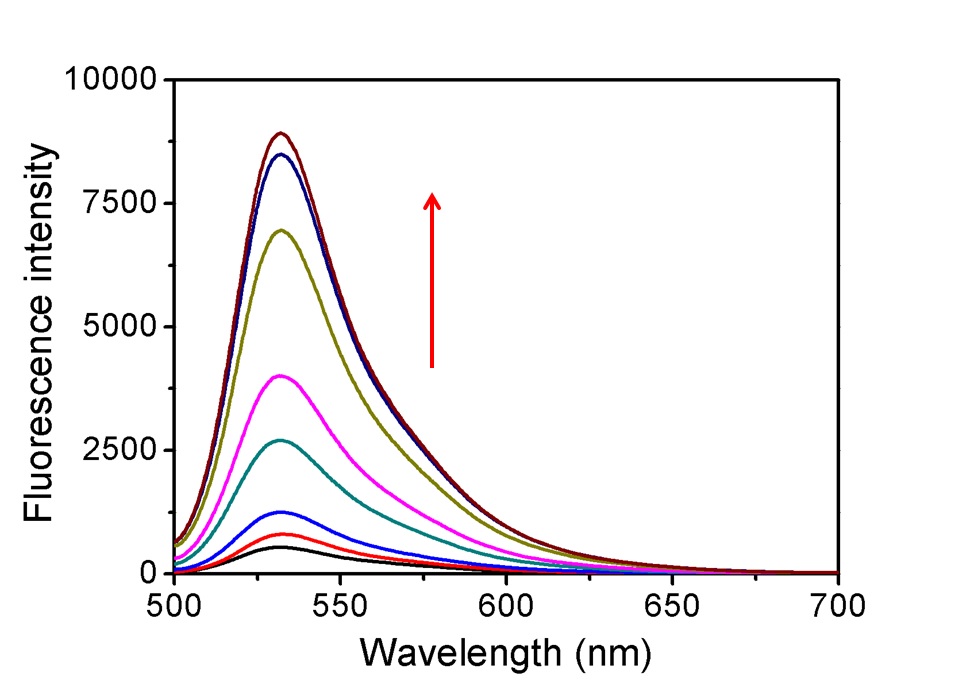


**Supplementary Figure 17.** The fluorescence spectra of SOSG solution (5.00 μM) containing the **MNPs** (the concentration of **TPP** was 500 nM) upon irradiation for different time (671 nm, 0.5 W cm^–2^).


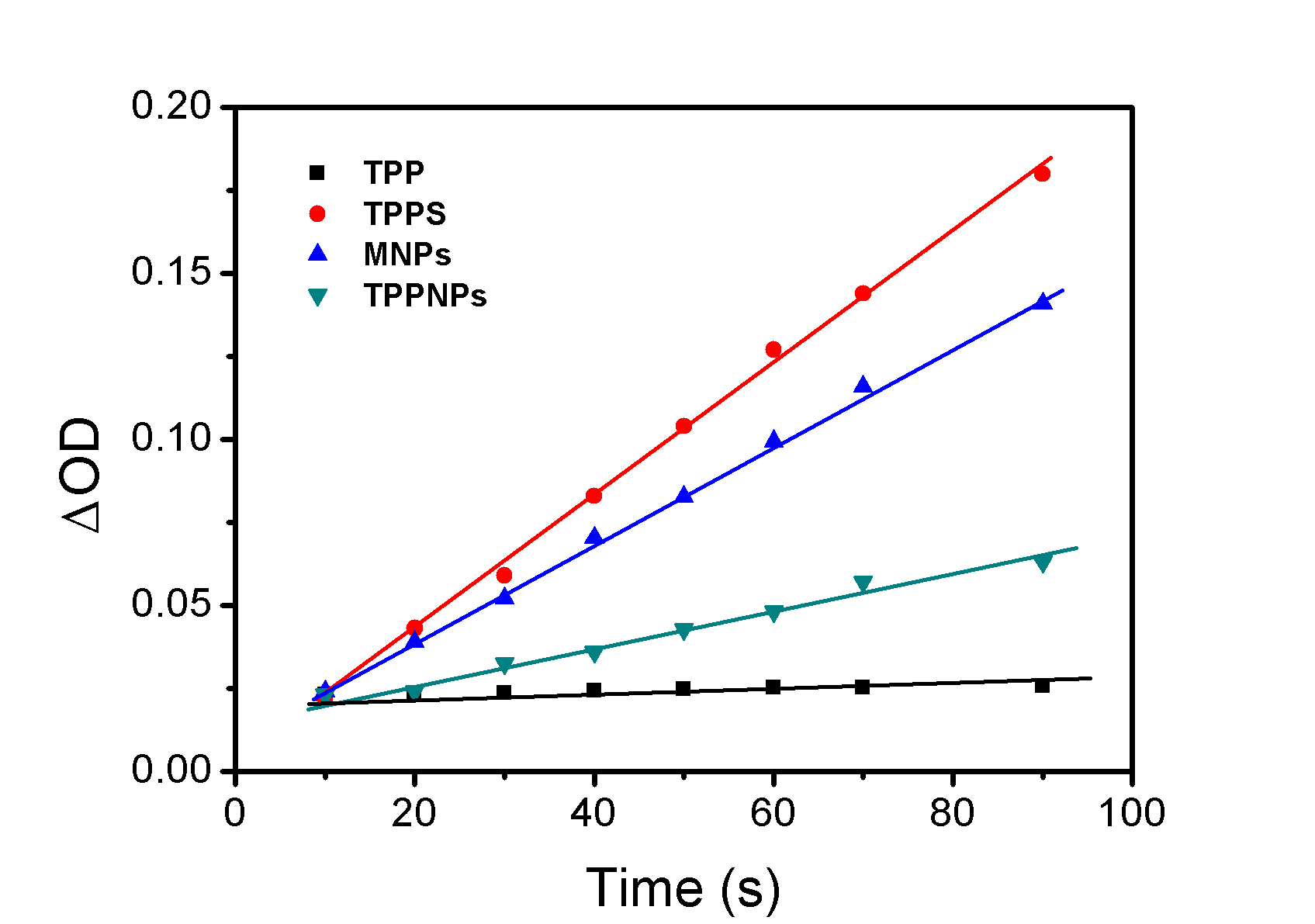


**Supplementary Figure 18*.*** Absorbance changes of DPBF at 415 nm *vs* irradiation time (671 nm, 0.5 W cm^–2^) in water in the presence of **TPP**, **TPPS**, **MNPs**, or **TPPNPs**. The concentration of the photosensitizers was 500 nM.

The changes in the absorption of DPBF was monitored at 415 nm as shown in Supplementary Figure 18, which is due to the dye sensitized generation of ^1^O_2_ followed by photo-oxidation of DPBF. Absorbance at the irradiating wavelength was adjusted to 0.02 for both the reference and **MNPs** (**TPP**, or **TPPNPs**). From the slope of the graph obtained by plotting change in optical density against the time interval, the ^1^O_2_ generation QY of **TPP**, **MNPs** and **TPPNPs** was calculated as 0.004, 0.44, and 0.12, respectively.

**
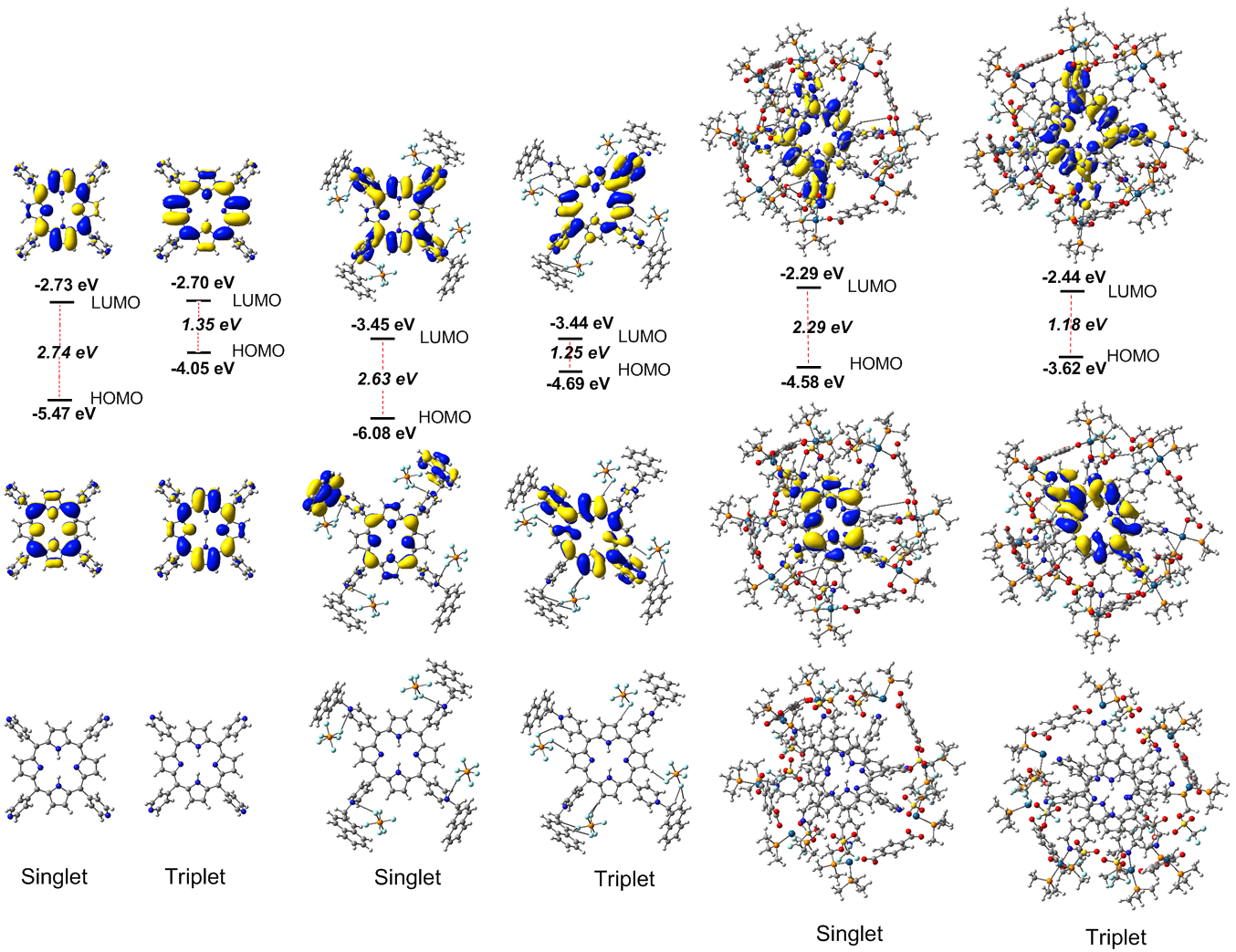
**

**Supplementary Figure 19*.*** TD-DFT result of **TPP** and **TPPN**.

*In vitro photochemotherapeutic investigations*


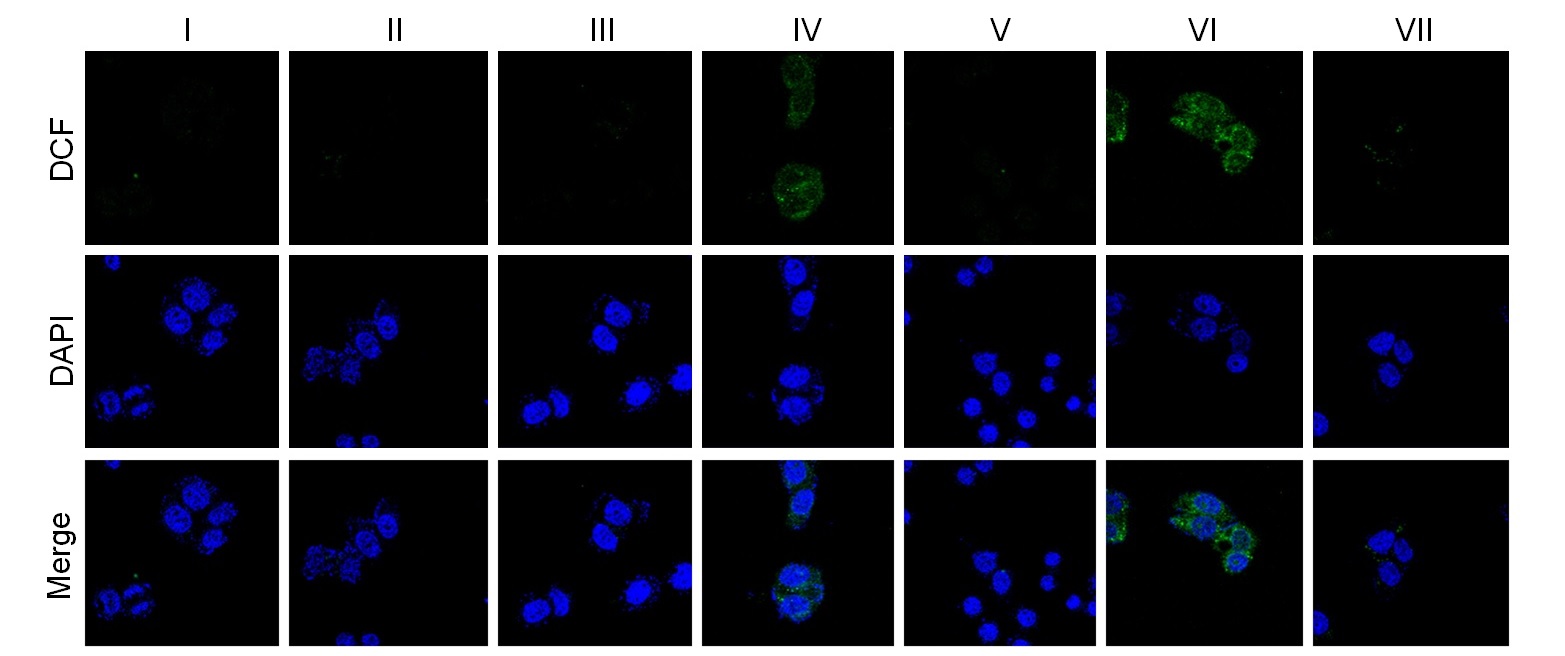


**Supplementary Figure 20*.*** Detection of intracellular ROS production for U87MG cells upon incubation with (I) ***c*Pt**; (II) cisplatin; (III) **MNPs**; (IV) **TPPNPs** + L; (V) **TPPNPs** + L in the presence of ROS scavenger (VC, 50 μM); (VI) **MNPs** + L; (VII) M**NPs** + L in the presence of ROS scavenger (VC, 50 μM). Green: DCF fluorescence; blue: DAPI fluorescence. The irradiation density was 0.1 W cm^–2^ at 671 nm, and the irradiation time was 3 min.

**
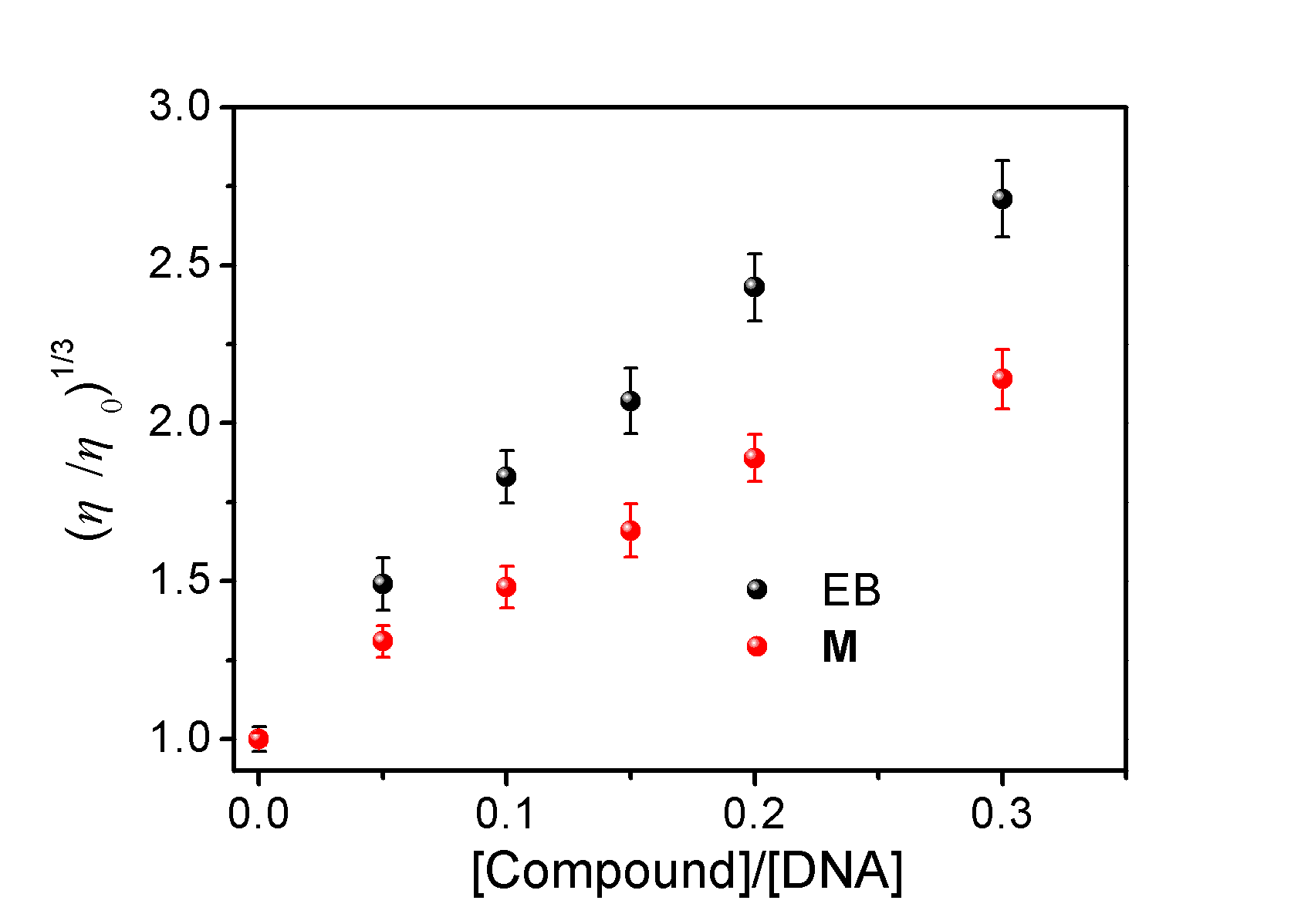
**

**Supplementary Figure 21*.*** Effect of addition of increasing quantities of **M** and the DNA intercalator ethidium bromide (EB) on the relative viscosity of ct-DNA (200 *μ*M) in PBS (pH = 7.2).


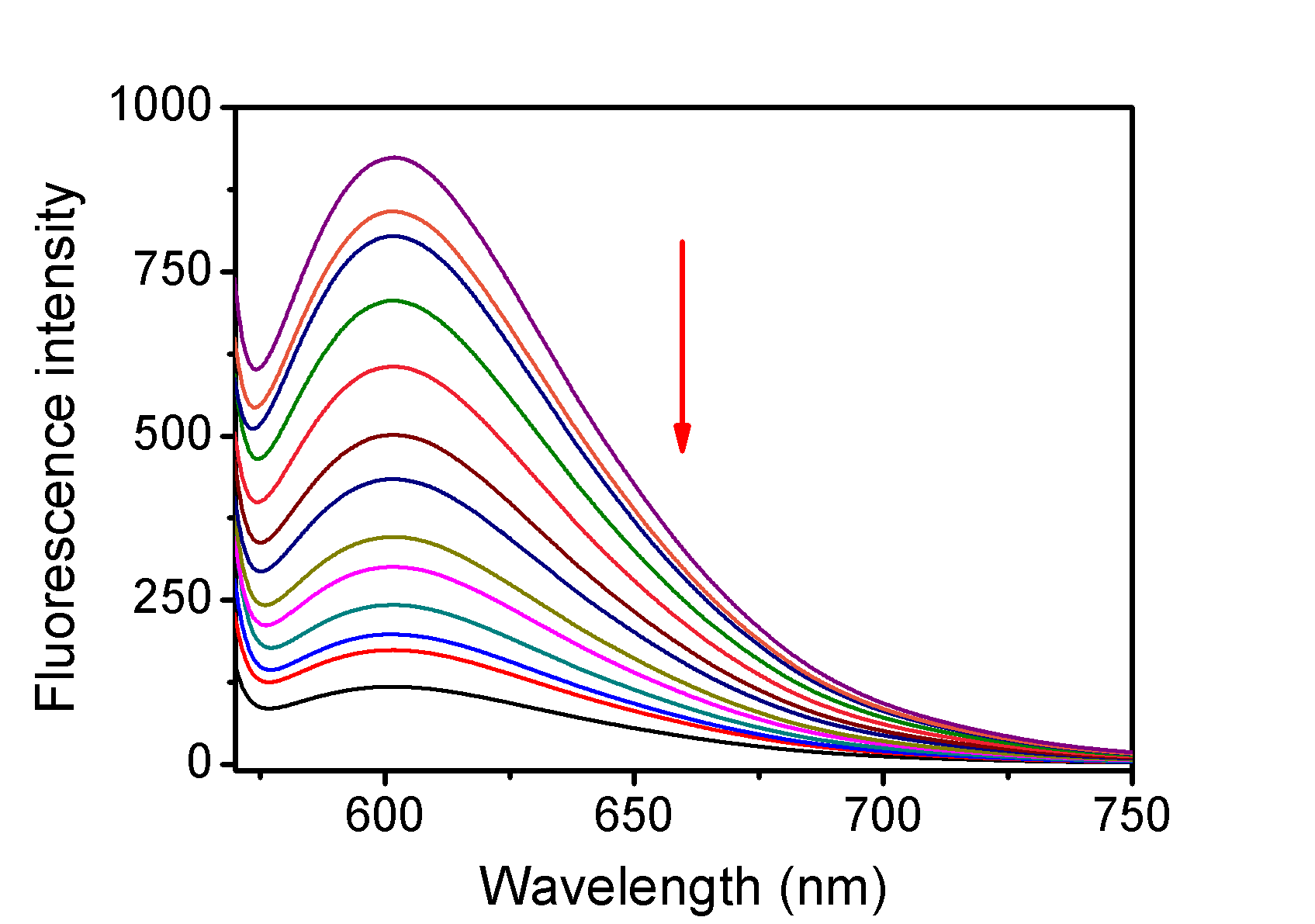


**Supplementary Figure 22*.*** Changes in emission spectrum of ct-DNA bound EB (*λ*_ex_ = 545 nm) with increasing concentration of **M** in PBS (pH = 7.4).

The cytotoxicity against U87MG cells was assessed by an MTT assay (Fig. 3f). The half-maximal inhibitory concentration (IC_50_) of ***c*Pt** was calculated to be 2.58 ± 0.4 *μ*M, comparable to that of cisplatin (IC_50_ = 2.24 ± 0.5 μM). The IC_50_ value of **MNPs** increased to 5.26 ± 0.78 μM in the dark. The IC_50_ value of the cells treated with **TPPNPs** was measured to be 0.41 ± 0.04 μM upon light irradiation at 671 nm for 3 min with the light density of 0.1 W cm^–2^. The most prominent results were observed with the **MNPs** showing an IC_50_ value of 21.4 ± 2.7 nM upon irradiation, which was much lower than those of chemotherapy (cisplatin, ***c*Pt**) and PDT (**TPPNPs**) alone. Interestingly, the mixture of ***c*Pt** and **TPPNPs** (4:1 in molar ratio) did not show a noticeable improvement in cytotoxicity (IC_50_ = 0.67 ± 0.09 μM, based on the molar amount of ***c*Pt**) upon irradiation, underlining that the formation of **M** played a remarkable role in the synergistic photochemotherapy.

Platinum-based chemotherapies mainly cause cytotoxicity by inducing apoptosis due to the formation of intra- and inter-strand crosslinks on DNA through coordination of the N_7_ atoms on the purine bases to the platinum centers, while PDT causes cytotoxicity through both apoptosis and necrosis pathways ascribing to the oxidation of DNA or proteins. The interactions between Pt and DNA were verified by viscometry and fluorescence titration experiments by using ethidium bromide (EB) as a control, a classic DNA intercalator that can lengthen and stiffen the calf-thymus DNA (ctDNA). Similar to that of EB, an enhancement in the relative viscosity of the solution containing ctDNA was observed as the concentration of **M** increased (Supplementary Figure 21), which was an indicator for the formation of ctDNA/Pt coordination complex. Moreover, a fluorescence EB-displacement titration showed that the fluorescence intensity of the ctDNA/EB complex decreased by gradually adding **M** (Supplementary Figure 22), indicative of the competitive binding of **M** to ctDNA.


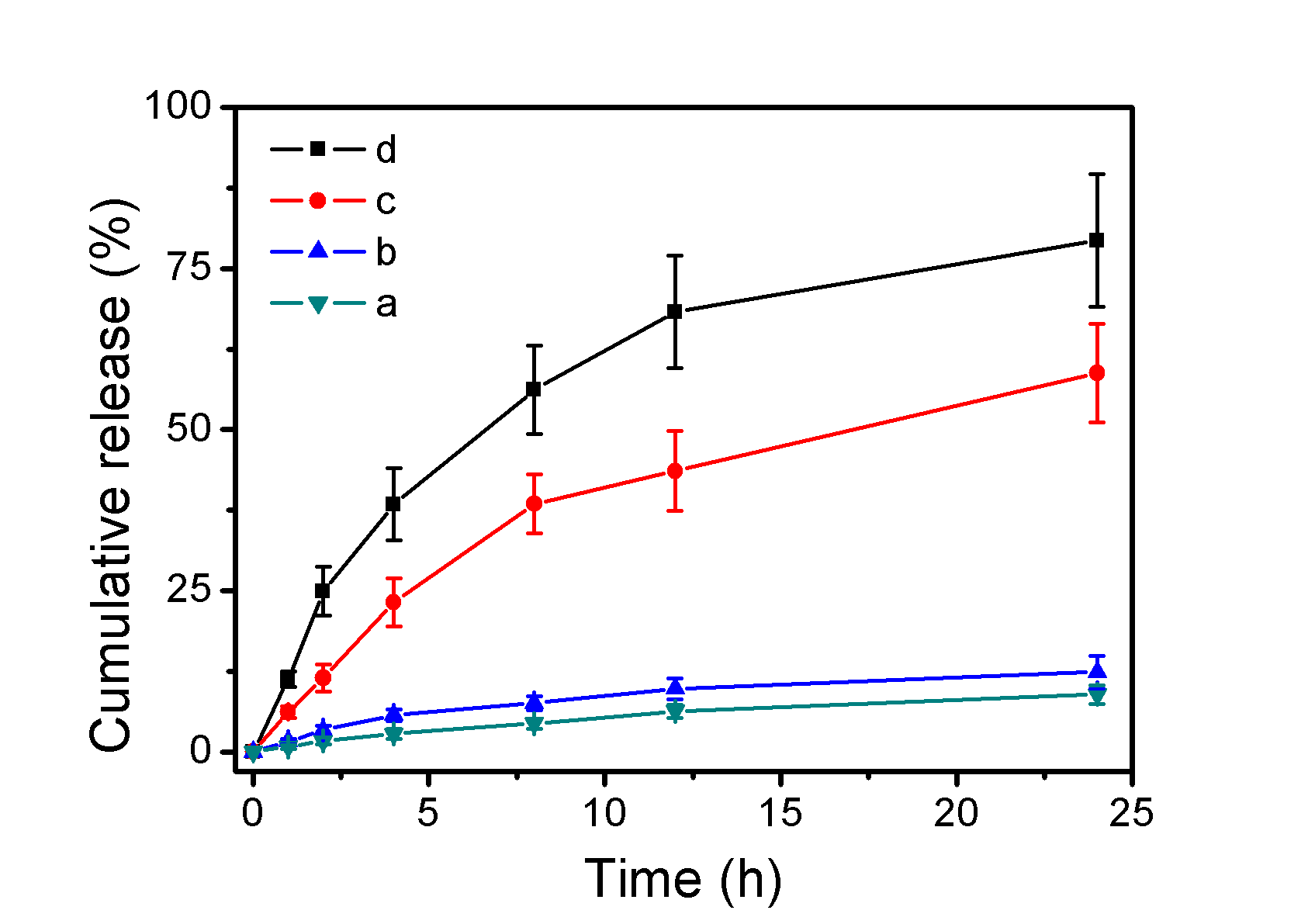


**Supplementary Figure 23*.*** Release profiles of the metallacage from **MNPs** under different conditions: (a) PBS (pH = 7.4), (b) PBS (pH = 7.4) + 10% FBS, (c) PBS (pH = 6.0) + 10% FBS, (d) PBS (pH = 5.0) + 10% FBS.

*In vivo theranostic studies*


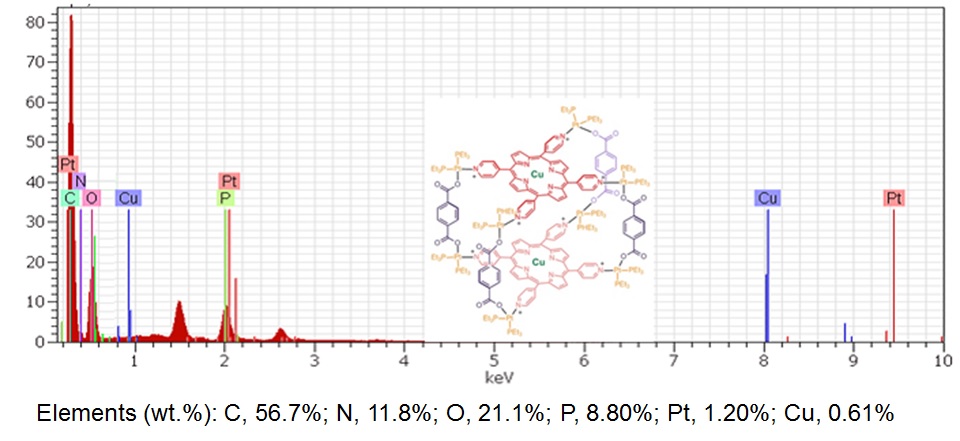


**Supplementary Figure 24.** EDS study of the Cu@**MNPs**.

**Supplementary Figure 25.** Radio TLC chromatograms of ^64^Cu@**MNPs** after labeling.


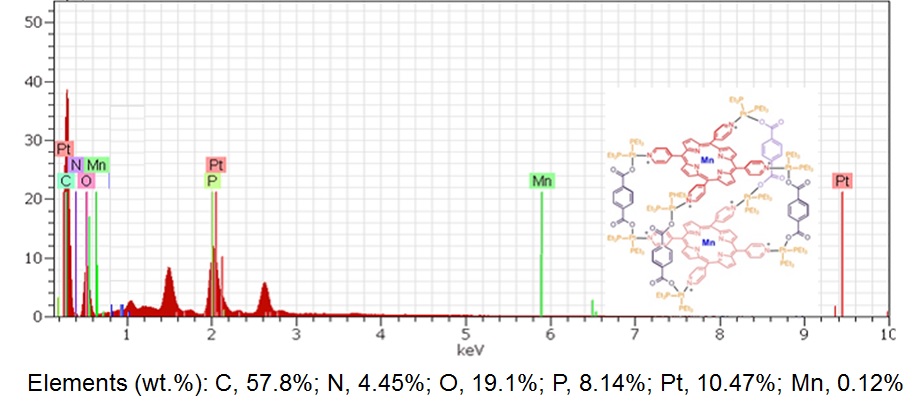


**Supplementary Figure 26.** EDS study of the Mn@**MNPs**.


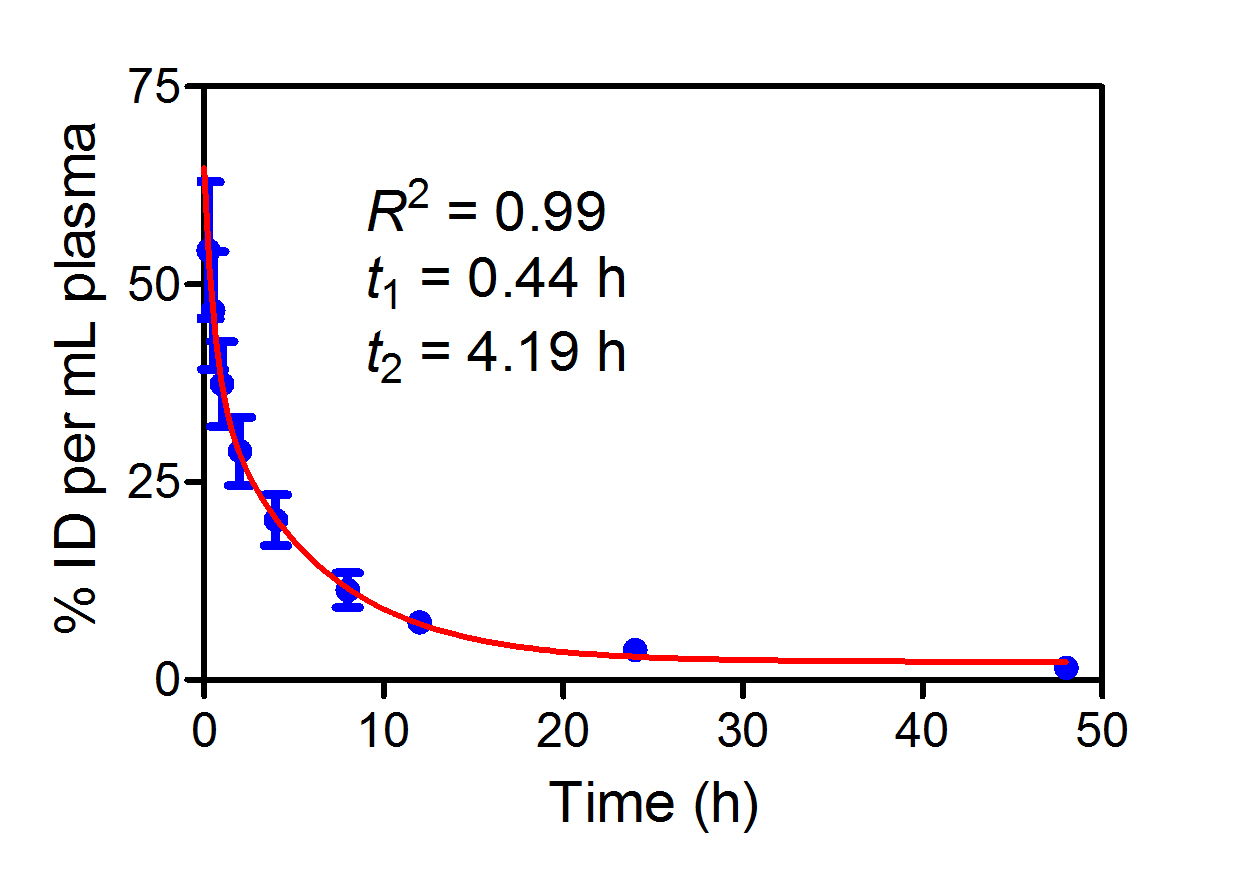


**Supplementary Figure 27.** Plasma platinum concentration versus time after injection of **MNPs** (2 mg Pt per kg) (*n* = 4).


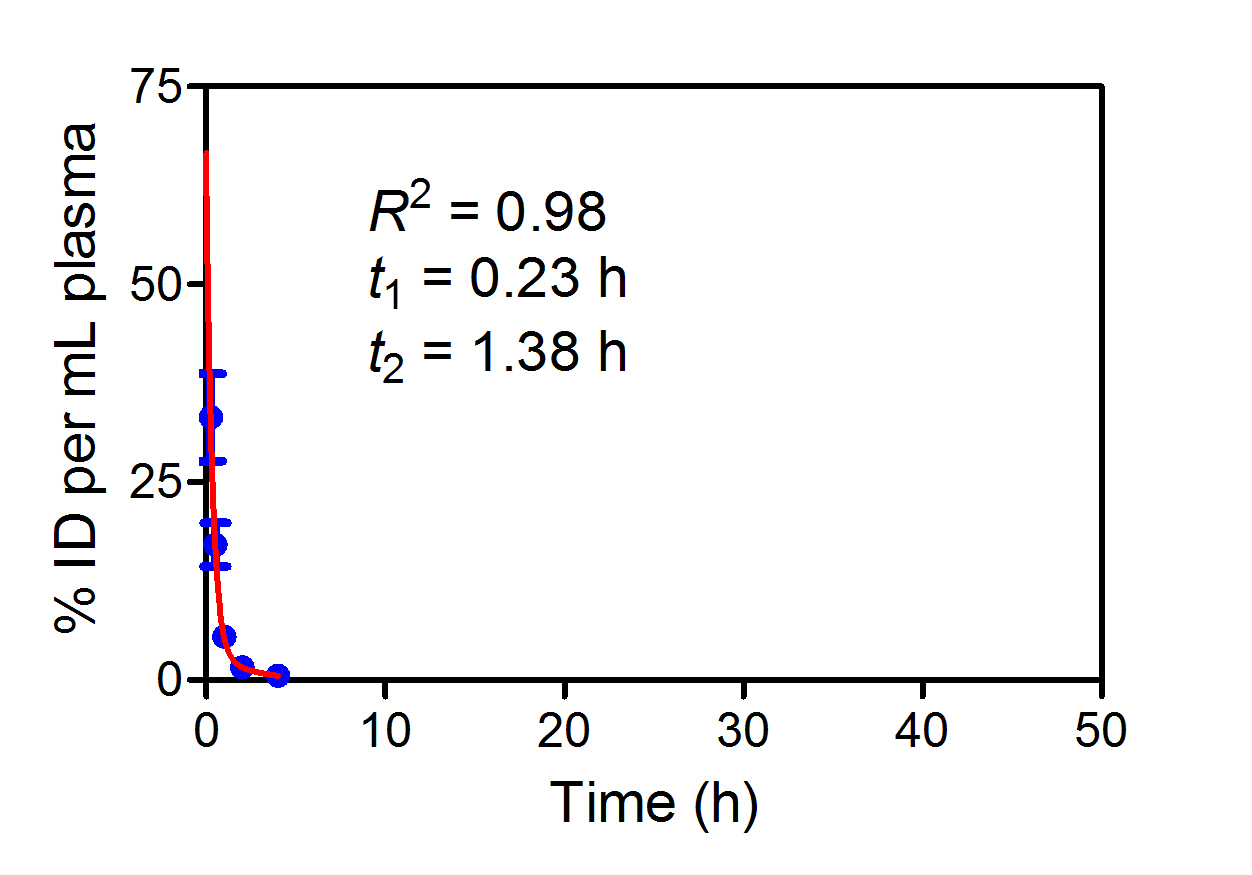


**Supplementary Figure 28.** Plasma platinum concentration versus time after injection of ***c*Pt** (2 mg Pt per kg) (*n* = 4).

The distribution half-life (*t*_1_) and elimination half-life (*t*_2_) of ***c*Pt** and **MNPs** were obtained using a two-compartment fitting model (Supplementary Figure 27 and Supplementary Figure 28). The *t*_1_ and *t*_2_ values of **MNPs** were calculated to be 0.44 h and 4.19 h, which were much higher than that of ***c*Pt** (*t*_1_ = 0.23 h, *t*_2_ = 1.38 h), confirming that the circulation time of **MNPs** was improved by taking advantage of EPR effect and active targeting capability. Compared with some reported nanomaterials, such as PEG modified UCNPs, iron oxide NPs and graphene, the half-life of **MNPs** was comparable or even better than them.^S5^

*
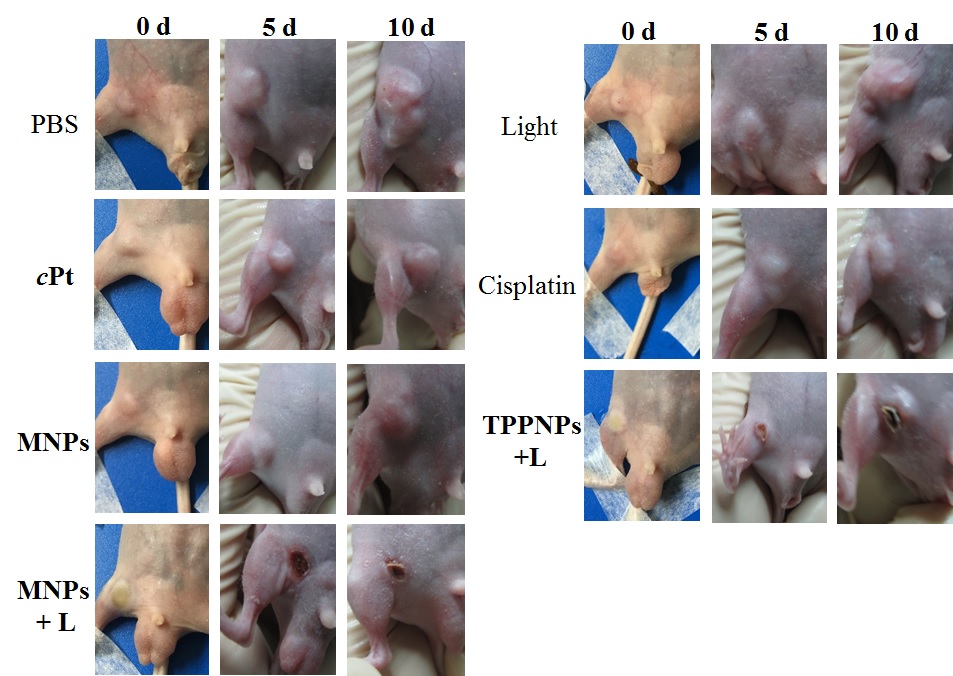
*

**Supplementary Figure 29.** Photographs of U87MG tumor-bearing mice after treatment with PBS, Light, ***c*Pt**, cisplatin, **MNPs**, **TPPNPs** + L, or **MNPs** + L. The irradiation density was 0.3 W cm^–2^ at 671 nm, and the irradiation time was 10 min.

*
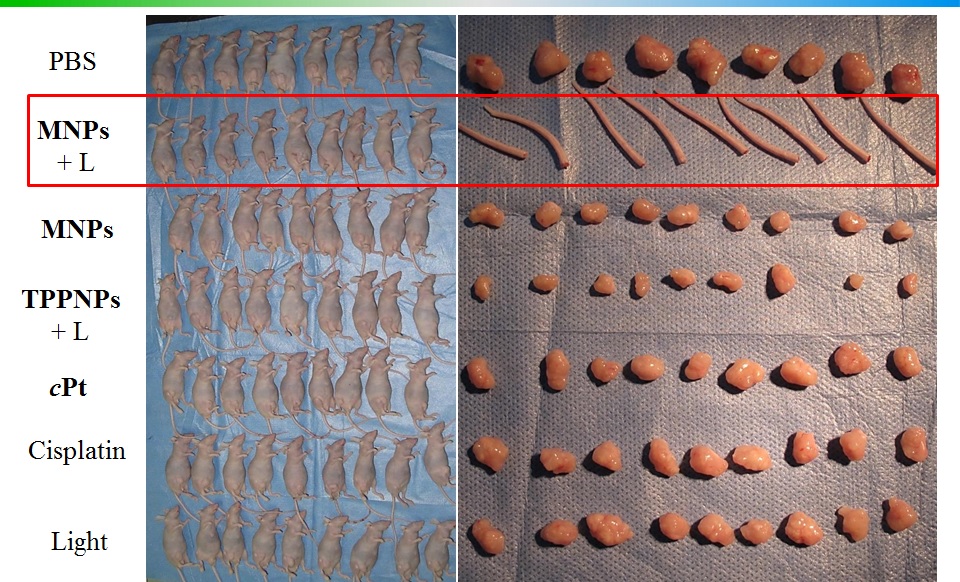
*

**Supplementary Figure 30.** Photographs of the mice and tumors harvested from the mice treated with PBS, Light, ***c*Pt**, cisplatin, **MNPs**, **TPPNPs** + L, or **MNPs** + L. The irradiation density was 0.3 W cm^–2^ at 671 nm, and the irradiation time was 10 min.

**
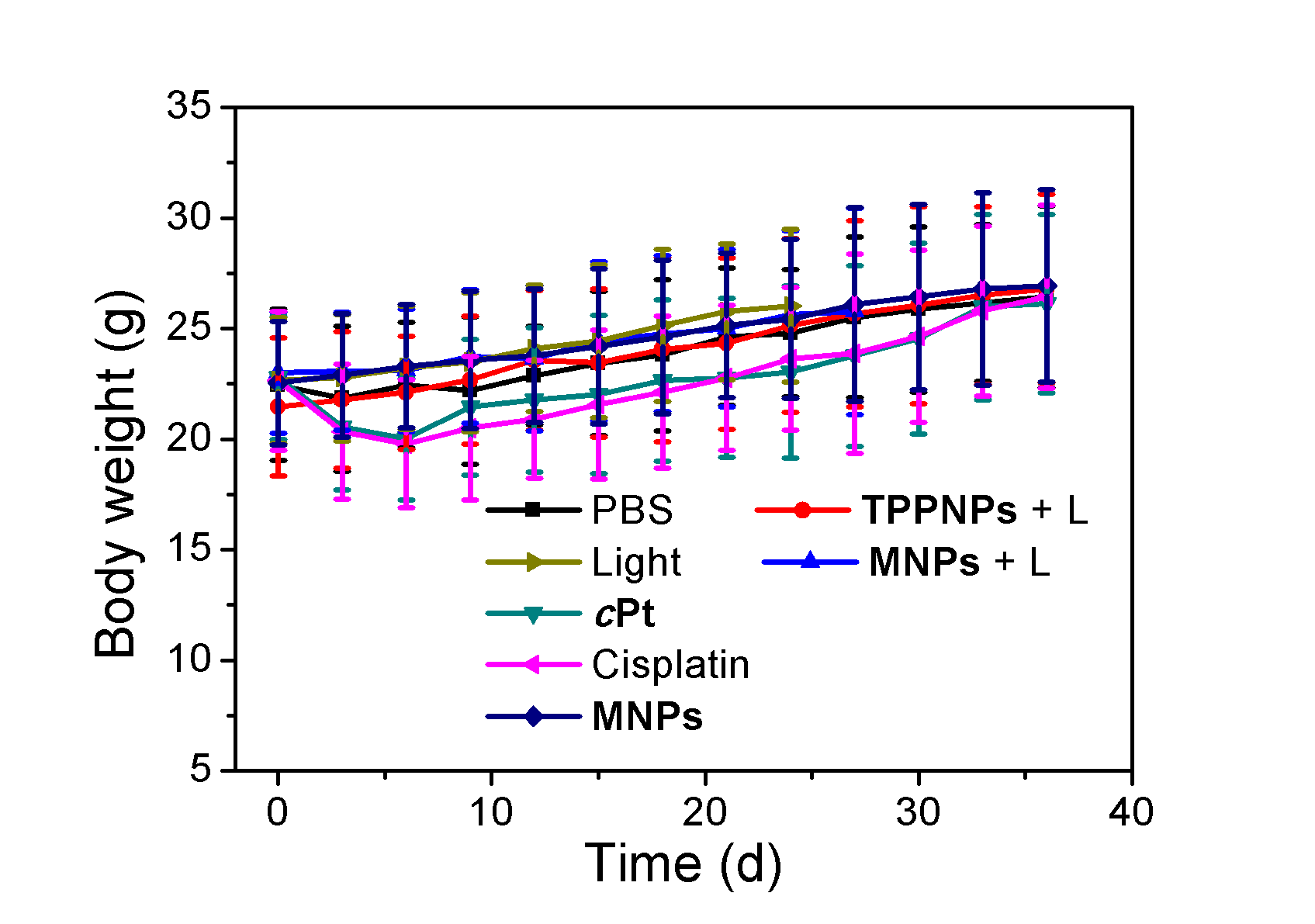
**

**Supplementary Figure 31.** Body weight changes of mice treated with PBS, Light, ***c*Pt**, cisplatin, **MNPs**, **TPPNPs** + L, or **MNPs** + L. The irradiation density was 0.3 W cm^–2^ at 671 nm, and the irradiation time was 10 min.


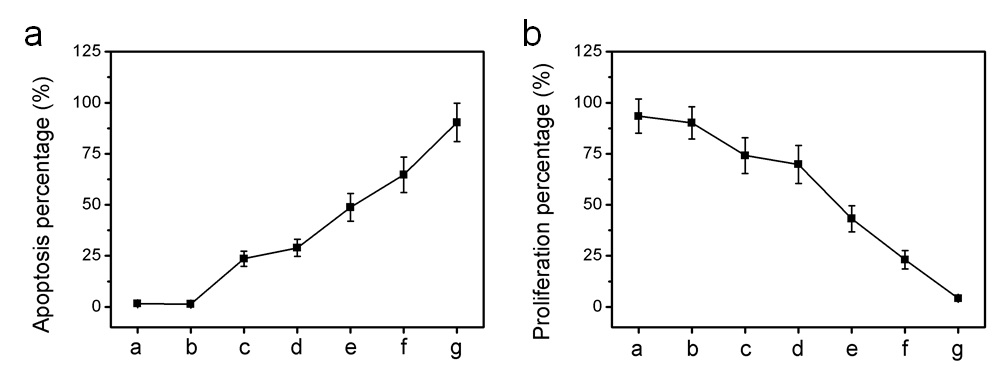


**Supplementary Figure 32.** Quantitative analysis of (a) tumor cell apoptosis and (b) cell proliferation of the mice treated with different formulations: a, PBS; b, Light; c, ***c*Pt**; d, cisplatin; e, **MNPs**; f, **TPPNPs** + L; g, **MNPs** + L.

*
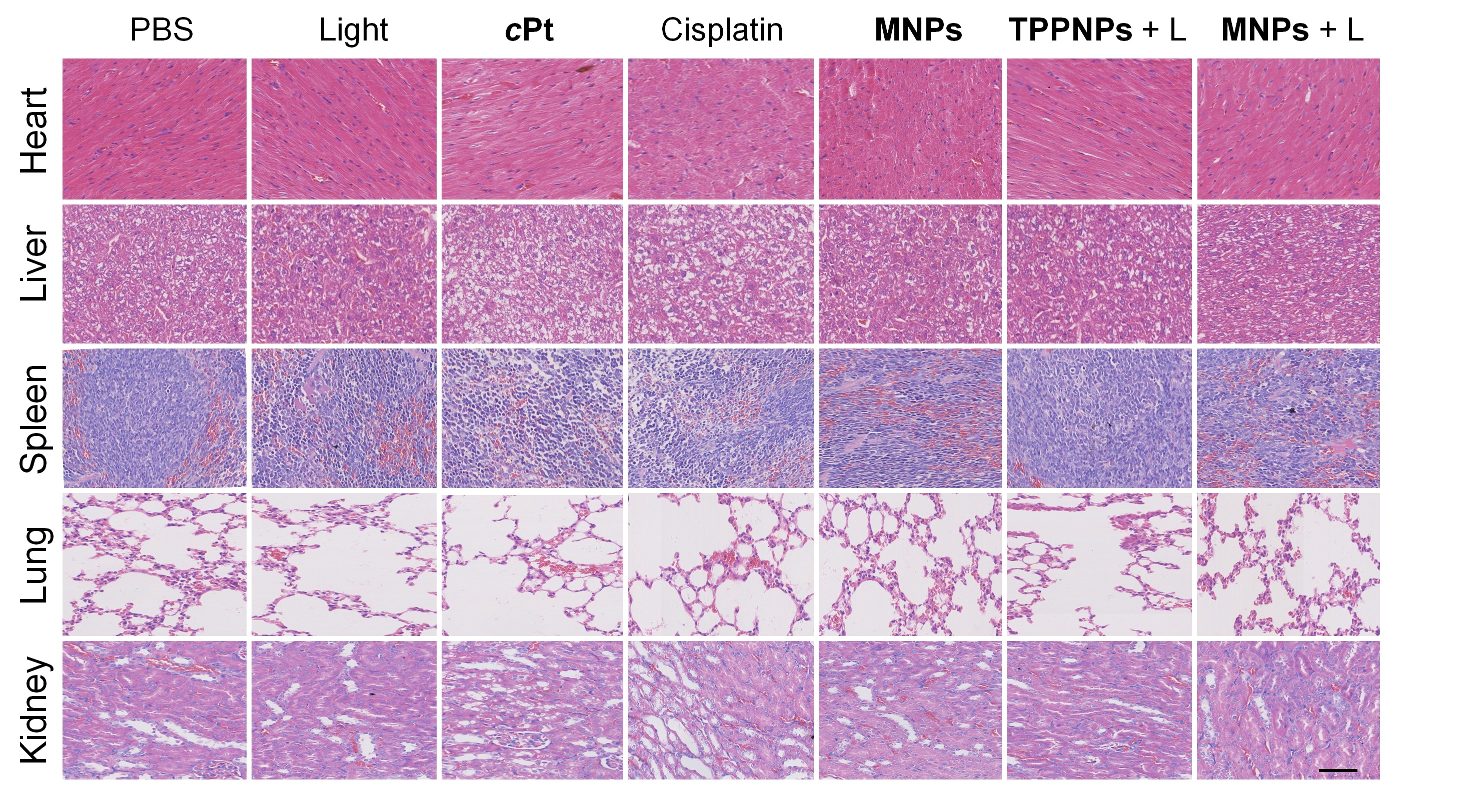
*

**Supplementary Figure 33.** H&E stained images of heart, liver, spleen, lung and kidney from different groups after treatment with PBS, light, ***c*Pt**, cisplatin, **MNPs**, **TPPNPs** + L, or **MNPs** + L. The irradiation density was 0.3 W cm^–2^ at 671 nm, and the irradiation time was 10 min. Scale bar is 100 μm.

**
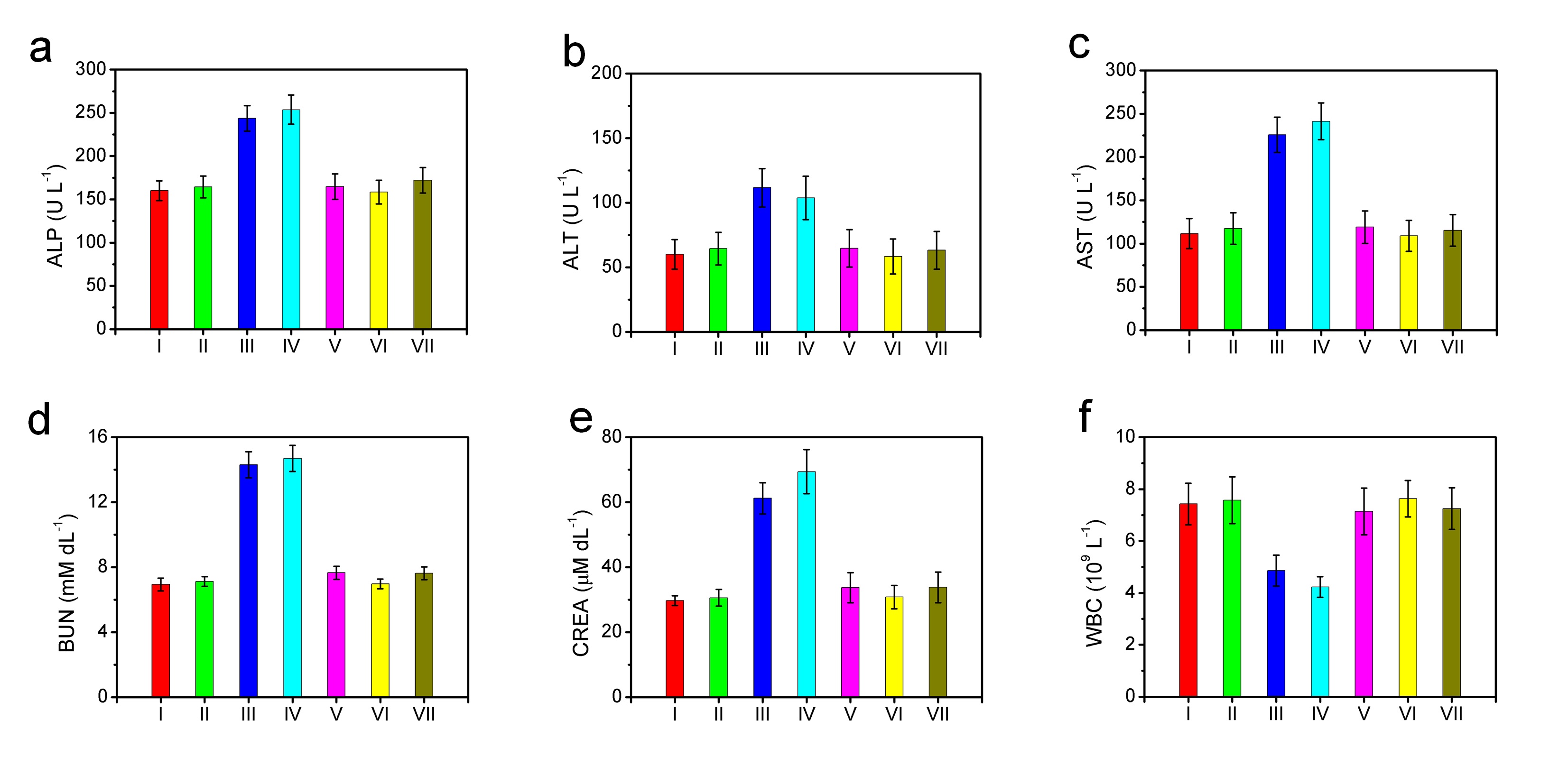
**

**Supplementary Figure 34.** Alterations of (a) ALP, (b) ALT, (c) AST, (d) BUN, (e) CREA, and (f) WBC in the mice bearing U87MG tumor xenograft (*n* = 6 for each group) at 3 days post-injection with (I) PBS, (II) light, (III) ***c*Pt**, (IV) cisplatin, (V) **MNPs**, (VI) **TPPNPs** + L, (VII) **MNPs** + L. The irradiation density was 0.3 W cm^–2^ at 671 nm, and the irradiation time was 10 min. ALP, alkaline phosphatase; ALT, alanine transferase; AST, aspartate transferase; BUN, blood urea nitrogen; CREA, creatinine; WBC, white blood cells. Data are expressed as mean ± standard deviation.


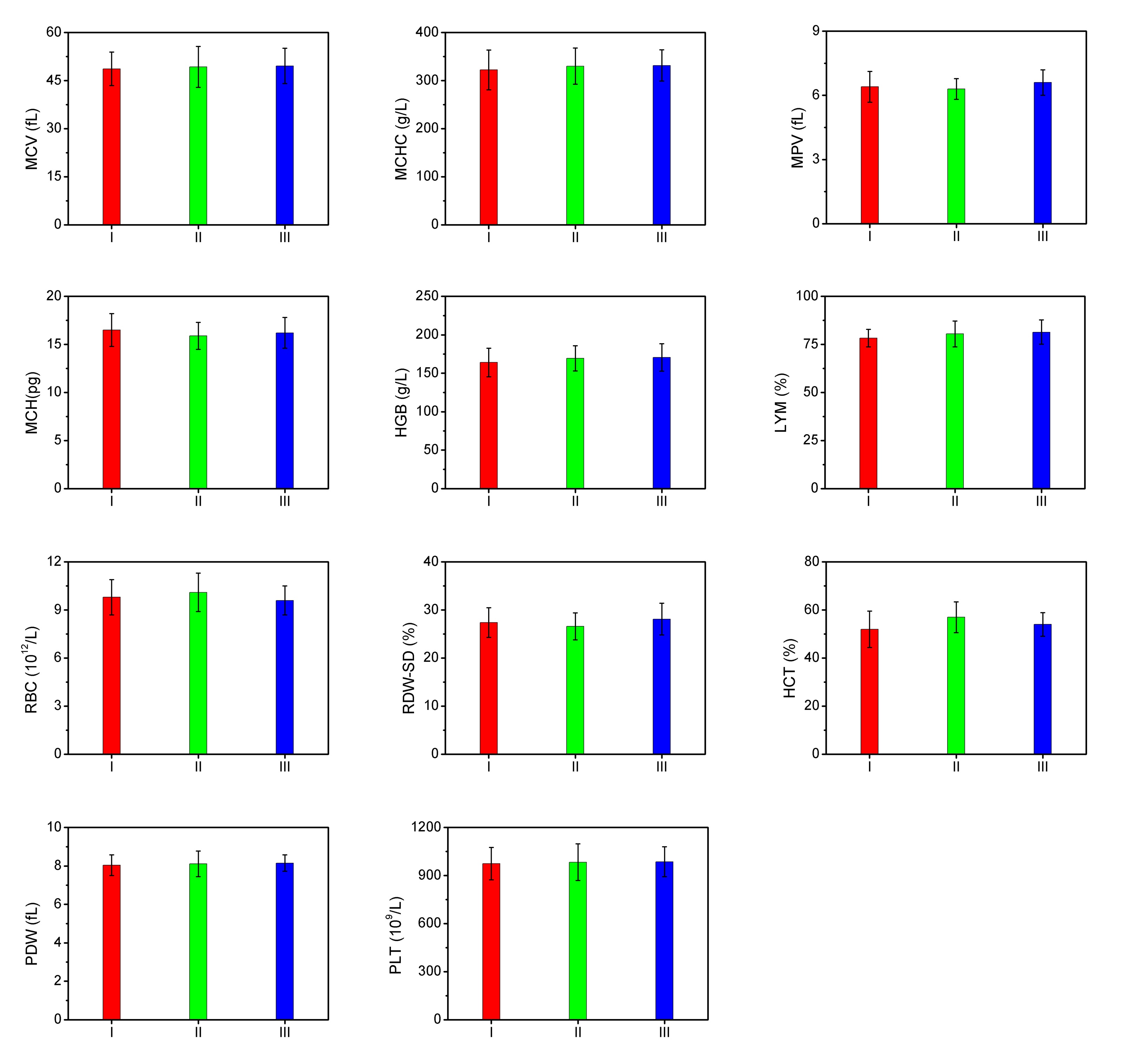


**Supplementary Figure 35.** Hematology analysis of the mice bearing U87MG tumor xenograft at 3 days post-injection with (I) PBS, (II) **MNPs**, or (III) **MNPs** + L. No significant changes with all tested parameters were detected. Red blood cells: RBC, platelets: PLT, mean corpuscular volume: MCV, mean corpuscular hemoglobin: MCH, mean corpuscular hemoglobin concentration: MCHC, hemoglobin: HGB, red cell distribution width standard deviation: RDW-SD, hematocrit: HCT, mean platelet volume: MPV, platelet distribution width: PDW, lymphocyte: LYM. Error bars represent standard deviation from 6 independent replicates.

To further evaluate the long-term potential toxicity of the **MNPs** *in vivo*, blood and urine were collected from the mice received different formulations for biochemistry assay. The level of clinical chemistry parameters including alkaline phosphatase, aspartate aminotransferase, alanine aminotransferase, creatinine, blood urea nitrogen, and creatinine were increased for the mice administrated with ***c*Pt** or cisplatin (Supplementary Figure 34), evidencing their severe hepatotoxicity and renal toxicity. Additionally, treatment with ***c*Pt** or cisplatin also led to the decrease in white blood cells after administration, an indicator of their systemic toxicity. Excitingly, no apparent changes were detected between the mice treated with **MNPs** (with or without laser irradiation) and PBS-treated mice in the liver function and renal function within the experimental period (Supplementary Figure 35). Other vital hematology markers, including red blood cells, platelets, hemoglobin, mean corpuscular volume, hematocrit, mean corpuscular hemoglobin, mean corpuscular hemoglobin concentration, red cell distribution width standard deviation, platelet distribution width, mean platelet volume, and lymphocyte all fell within normal ranges for the mice treated with **MNPs**, showing no noticeable toxicity and inflammatory response.


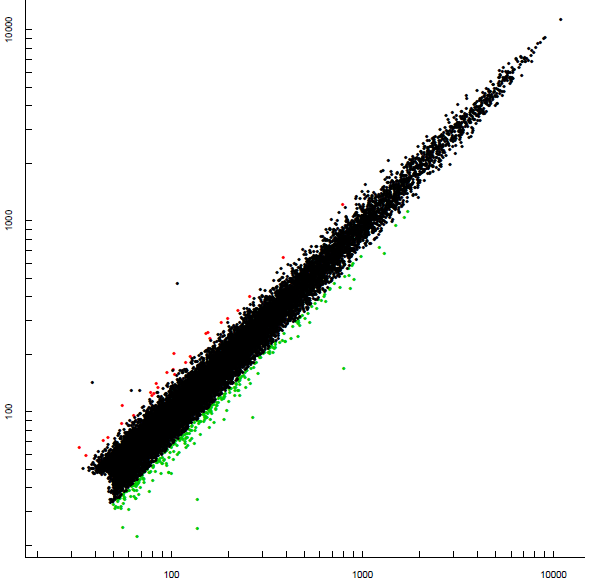


**Supplementary Figure 36.** Pearson correlations for the chemotherapy group (**MNPs**).


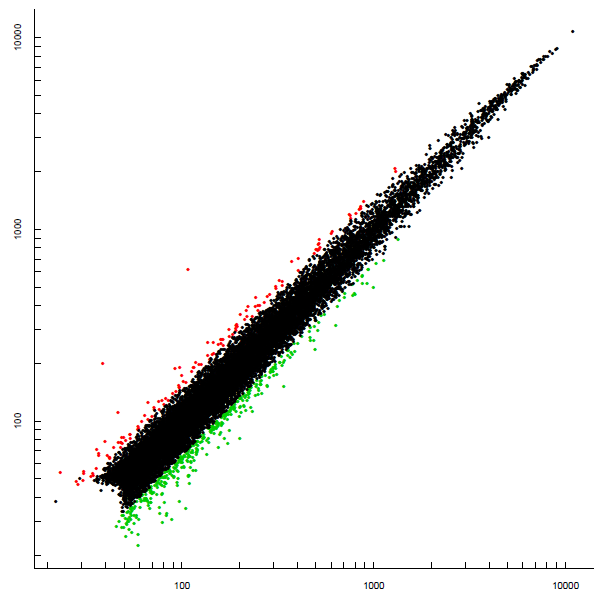


**Supplementary Figure 37.** Pearson correlations for the PDT group (**TPPNPs** + L)


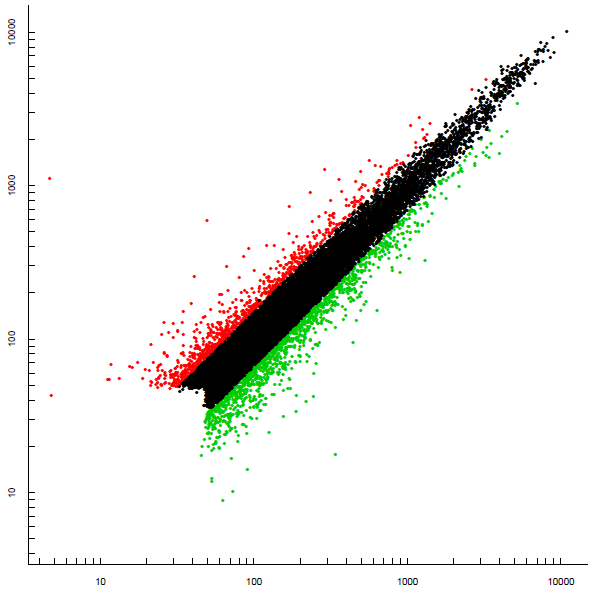


**Supplementary Figure 38.** Pearson correlations for the photochemotherapy group (**MNPs** + L).


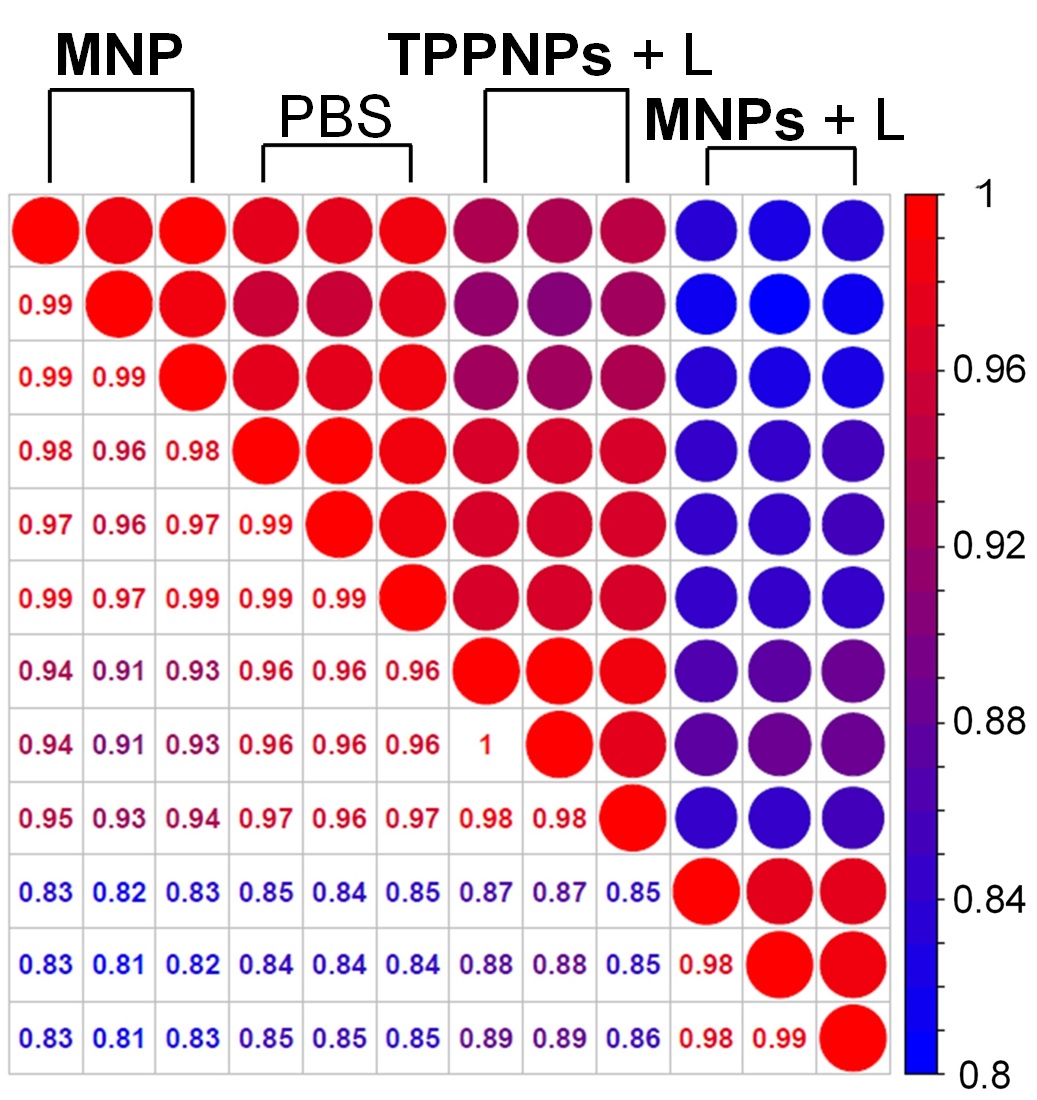


**Supplementary Figure 39.** Pearson correlation for triplicate microarray samples in treated (**TPPNPs** + L and **MNPs** + L) and controlled (PBS) mice. The whole genome data is representative from three different tumors.


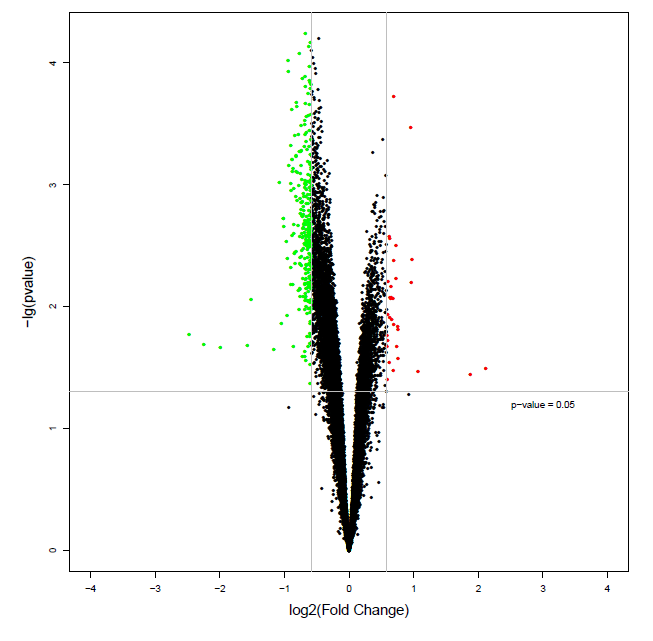


**Supplementary Figure 40.** Volcano plots for the chemotherapy group (**MNPs**).


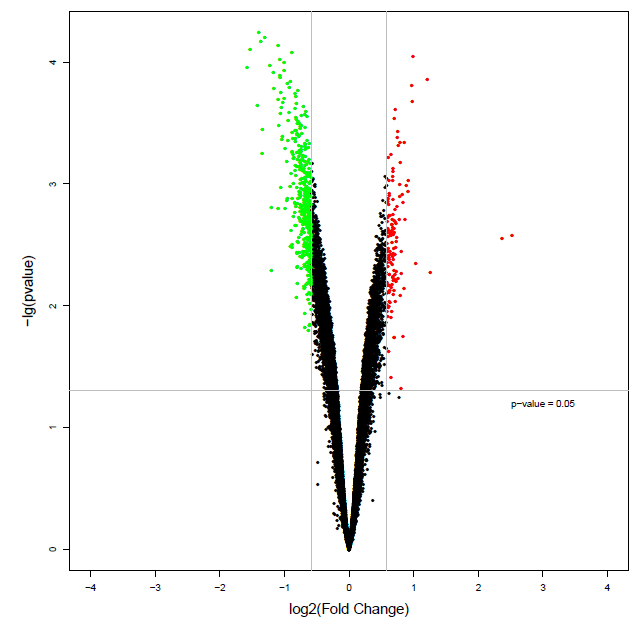


**Supplementary Figure 41.** Volcano plots for the PDT group (**TPPNPs** + L).


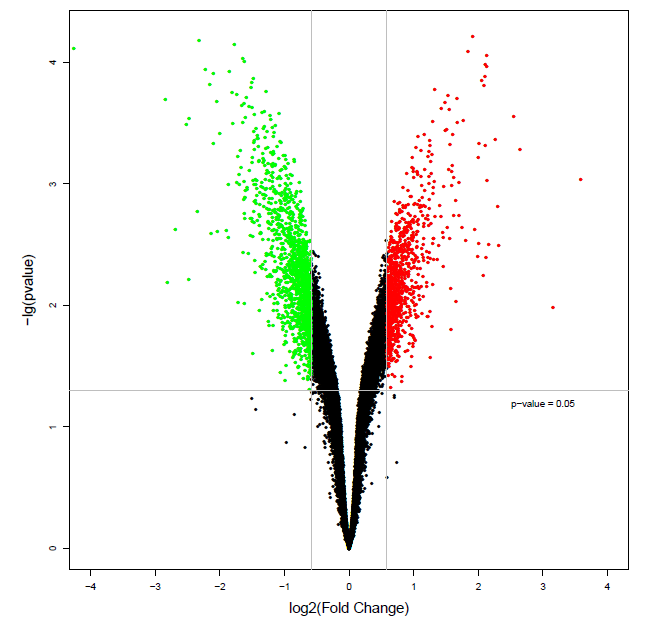


**Supplementary Figure 42.** Volcano plots for the photochemotherapy group (**MNPs** + L).


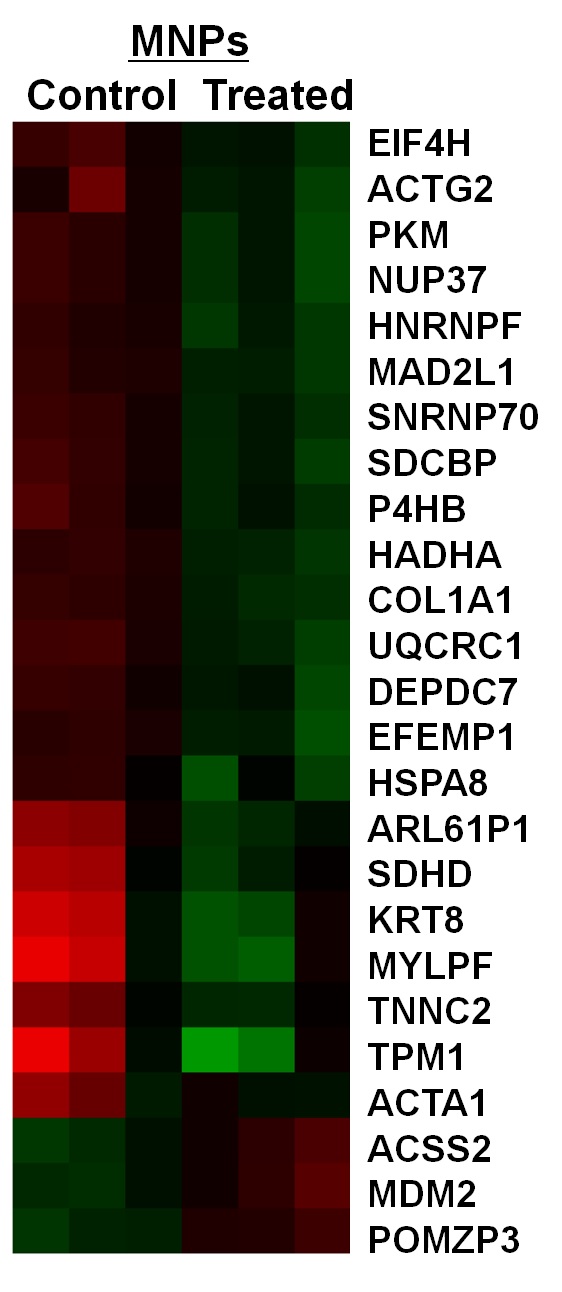


**Supplementary Figure 43.** The top 25 largest absolute fold change genes for the chemotherapy group (**MNPs**) compared with the controlled groups. The whole genome data is representative from three different tumors.


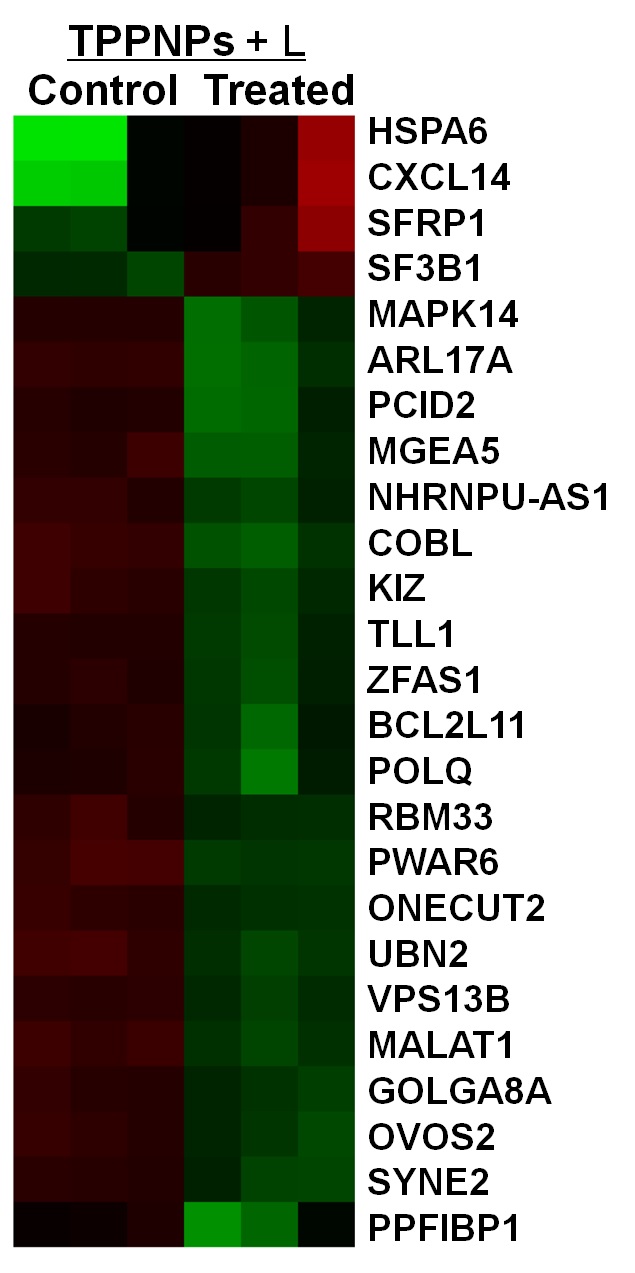


**Supplementary Figure 44.** The top 25 largest absolute fold change genes for the PDT group (**TPPNPs** + L) compared with the controlled groups. The whole genome data is representative from three different tumors.


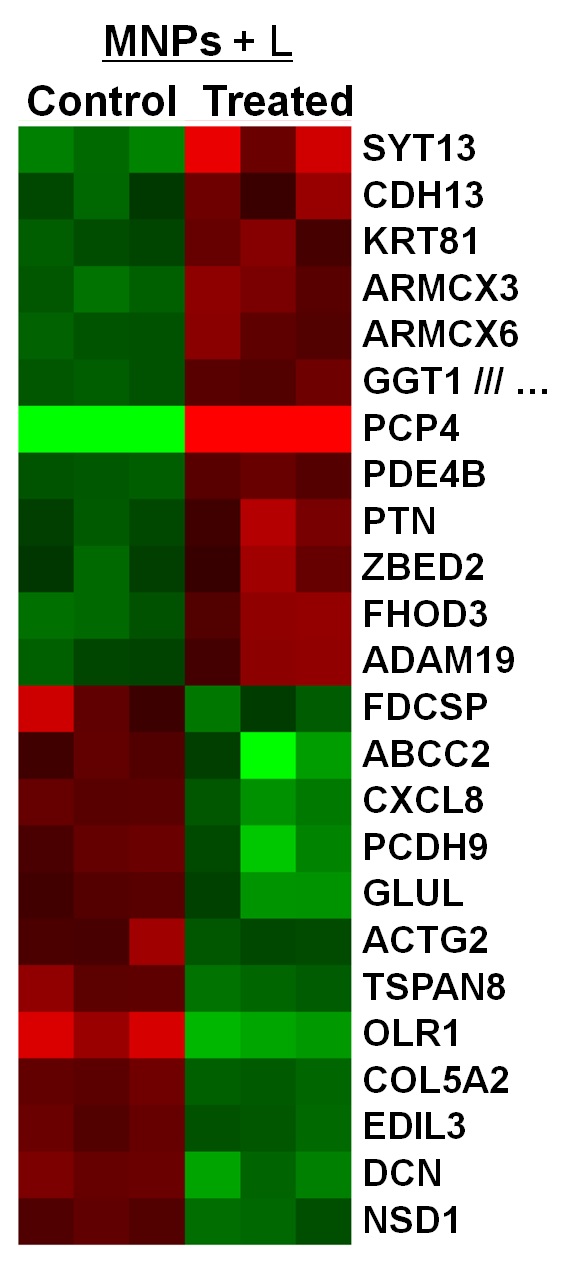


**Supplementary Figure 45.** The top 24 largest absolute fold change genes for the photochemotherapy group (**MNPs** + L) compared with the controlled groups. The whole genome data is representative from three different tumors.


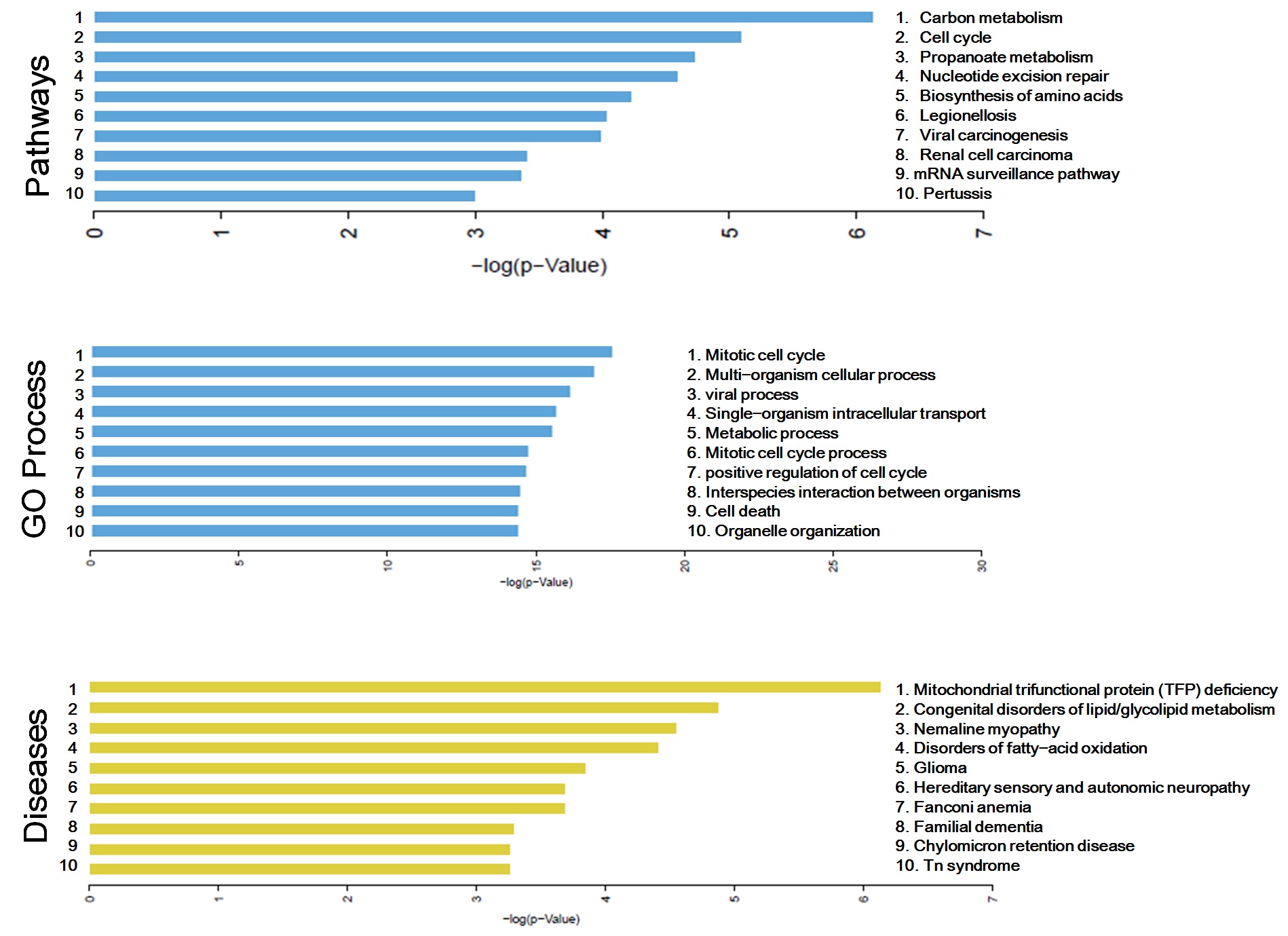


**Supplementary Figure 46.** Enrichment Analysis Workflow for altered genes exclusively on the chemotherapy group (**MNPs**), concerning Pathway Maps, Gene Ontology (GO) Processes and Diseases (by Biomarkers).


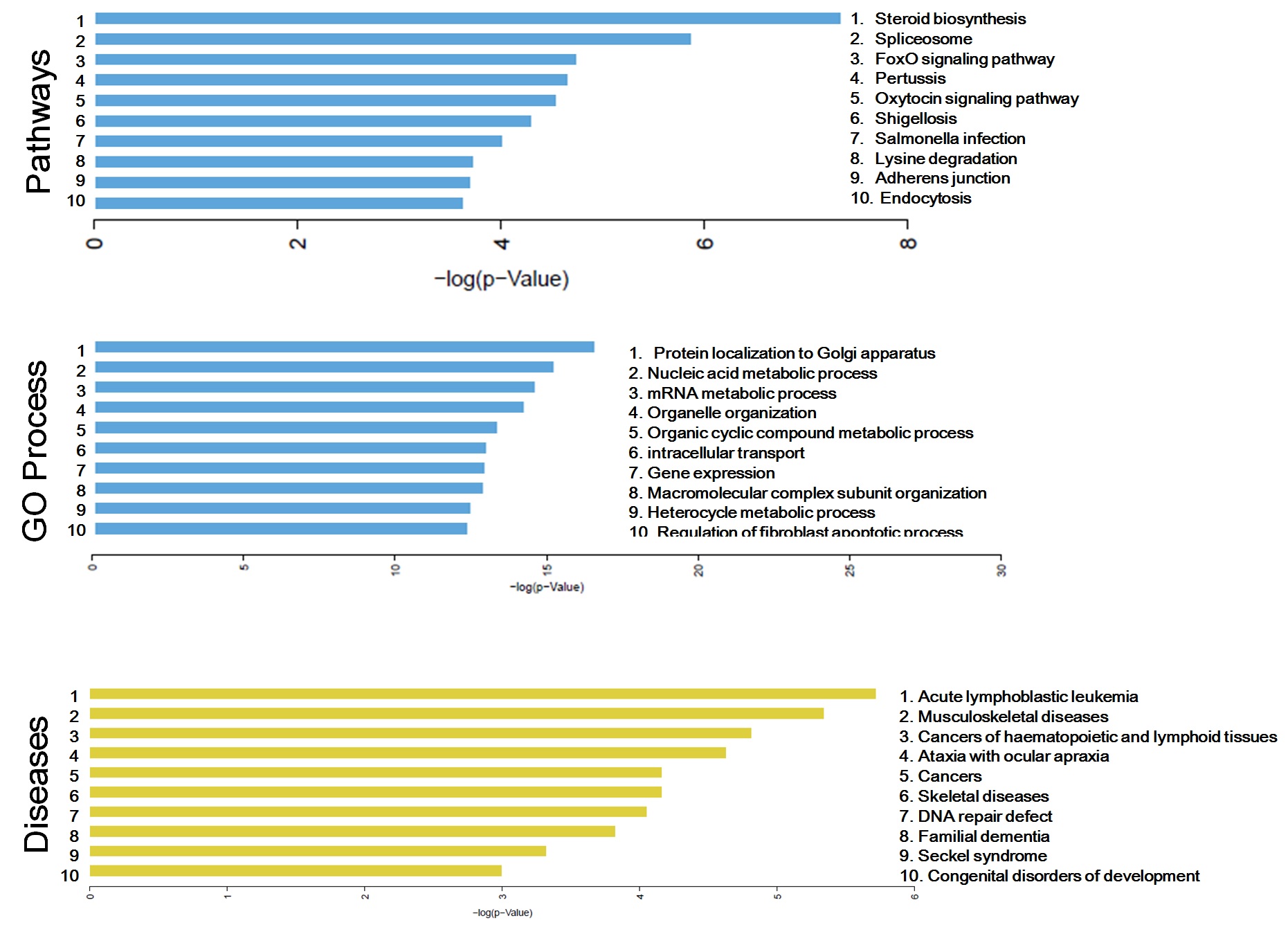


**Supplementary Figure 47.** Enrichment Analysis Workflow for altered genes exclusively on the PDT group (**TPPNPs** + L), concerning Pathway Maps, Gene Ontology (GO) Processes and Diseases (by Biomarkers).


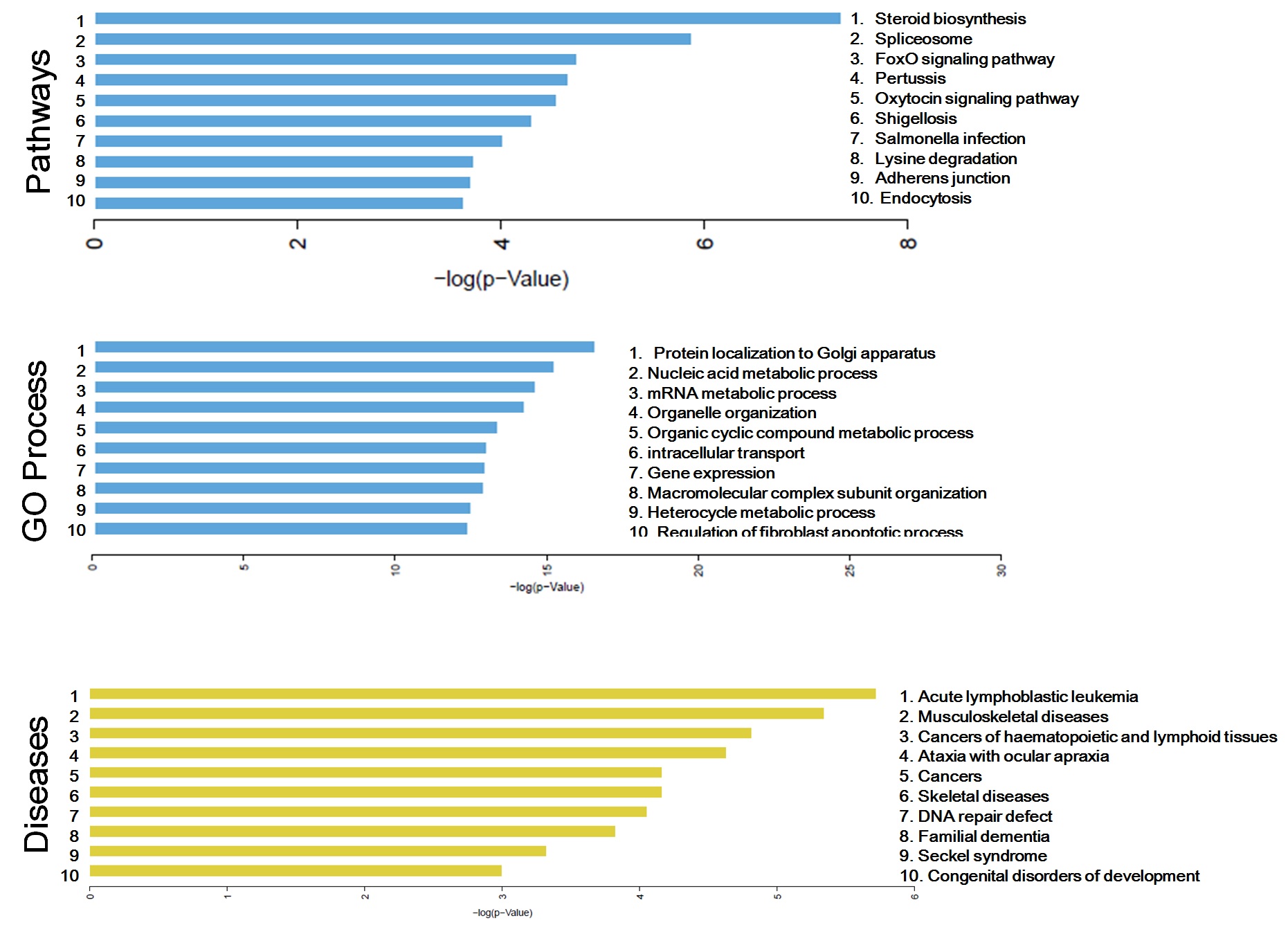


**Supplementary Figure 48.** Enrichment Analysis Workflow for altered genes exclusively on the photochemotherapy group (**MNPs** + L), concerning Pathway Maps, Gene Ontology (GO) Processes and Diseases (by Biomarkers).


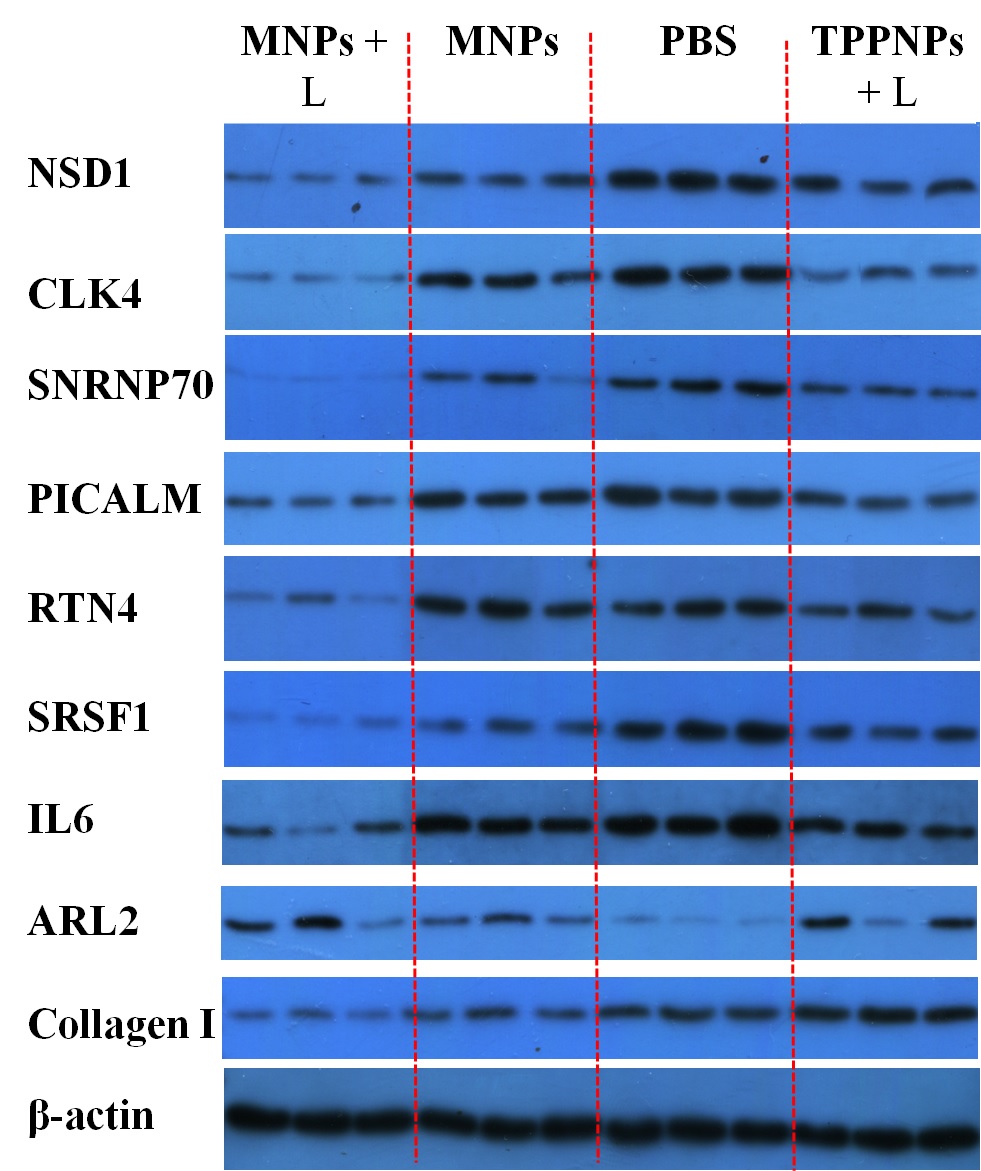


**Supplementary Figure 49.** Western blotting staining of selective proteins.

From MTT assay, the IC_50_ values of different formulations towards cisplatin resistant ovarian carcinoma (A2780CIS) were calculated to be 19.1 ± 2.77, 15.7 ± 2.61, and 27.2 ± 3.66 μM for ***c*Pt**, cisplatin, and **MNPs**, respectively (Fig. 7a), which were much higher than those towards non-resistant A2780 cell lines (2.30 ± 0.27, 1.74 ± 0.24, and 3.57 ± 0.46 μM for ***c*Pt**, cisplatin, and **MNPs**, respectively (Supplementary Figure 50). For **TPPNPs** + L, the IC_50_ values against A2780CIS (1.34 ± 0.26 μM) and A2780 (0.97 ± 0.12 μM) cell lines were comparable because of different anticancer mechanism between chemotherapy and PDT. The most promising results were observed with the **MNPs** upon irradiation, the cytotoxicity towards A2780CIS cell was improved by a factor of 110 for the **MNPs**, yielding an IC_50_ value of 247 ± 34.3 nM. As a control, the anticancer result was less effective by simply mixing the ***c*Pt** and **TPPNPs** (4:1, molar ratio) followed by irradiation, implying the significant role in synergistic effect by integrating the porphyrin and ***c*Pt** into one platform.


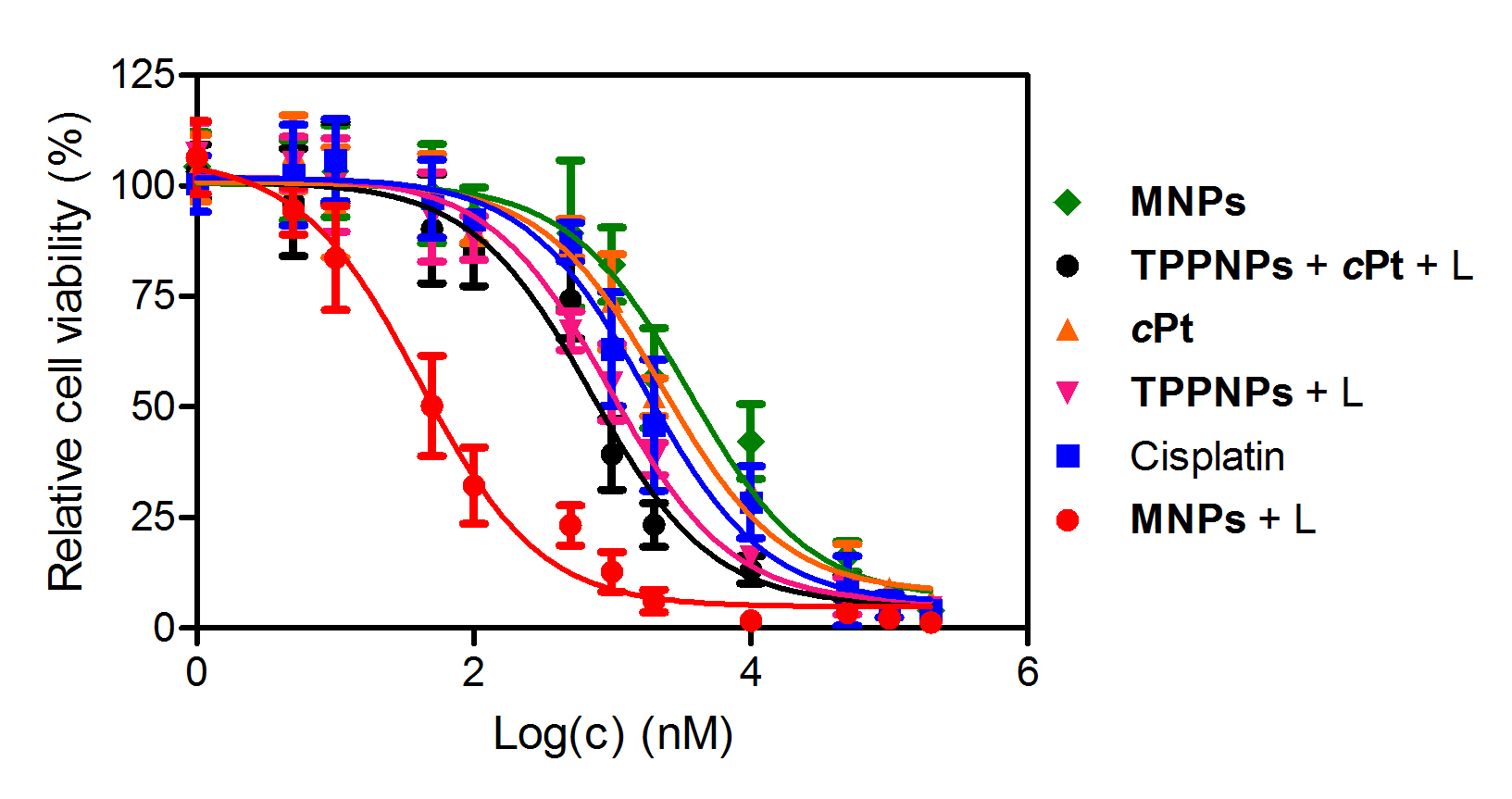


**Supplementary Figure 50.** Cytotoxicity evaluation of A2780 cells treated with different administrations. The irradiation density was 0.1 W cm^–2^ at 671 nm, and the irradiation time was 3 min.


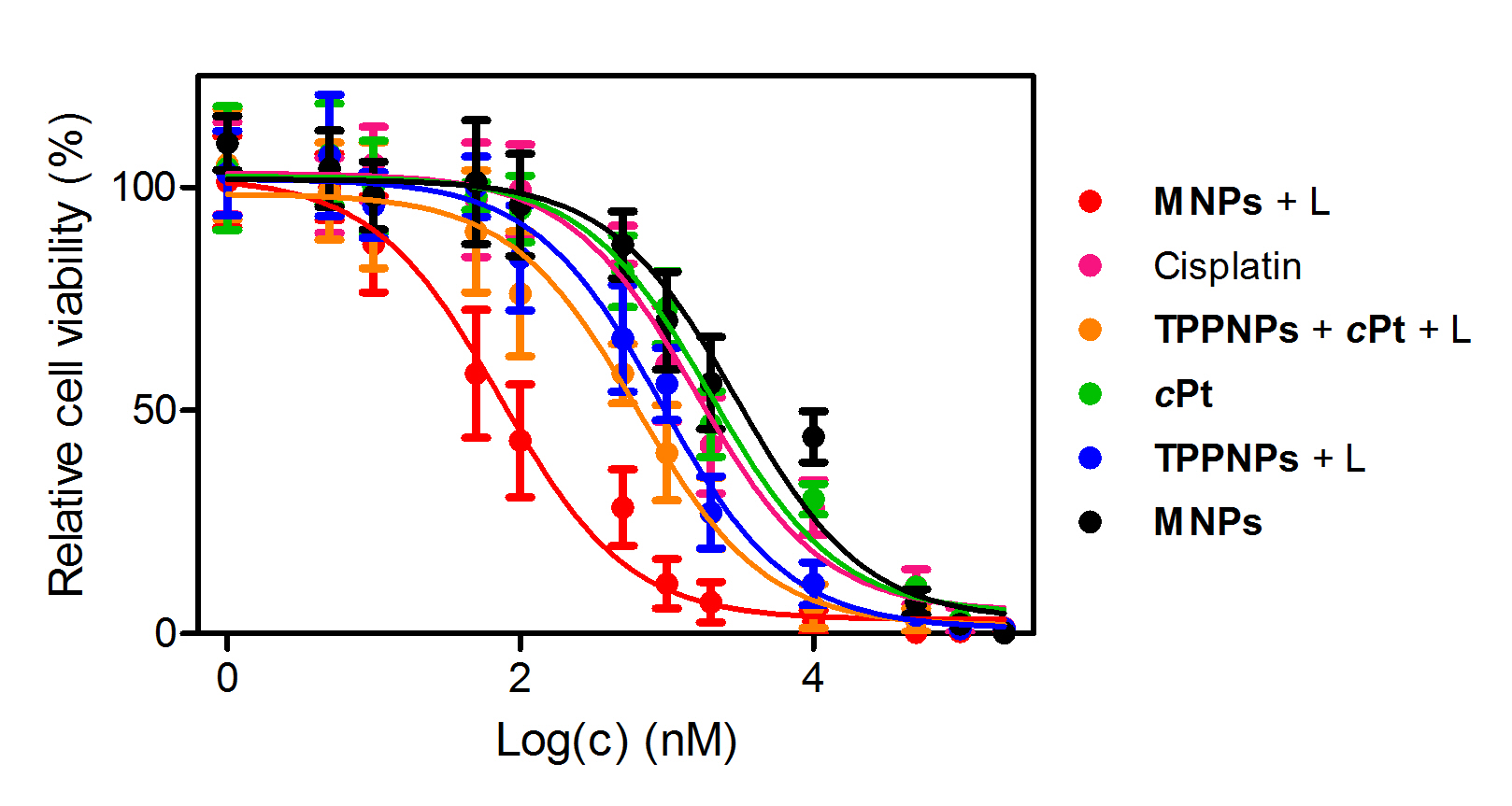


**Supplementary Figure 51.** Cytotoxicity evaluation of 4T1 cells treated with different administrations. The irradiation density was 0.1 W cm^–2^ at 671 nm, and the irradiation time was 3 min.

From MTT assay (Supplementary Figure 51), the IC_50_ value was calculated to be 73.4 ± 3.97 nM for the 4T1 cells treated with **MNPs** followed by 3 min laser irradiation (671 nm, 0.1 W cm^–2^), which was much lower than those of cisplatin (1.58 ± 0.17 μM), ***c*Pt** (1.99 ± 0.25 μM), **MNPs** (3.01 ± 0.38 μM), and **TPPNPs** + L (IC_50_ = 915 ± 78.4 nM), demonstrating its superior synergistic anticancer efficacy *in vitro*.


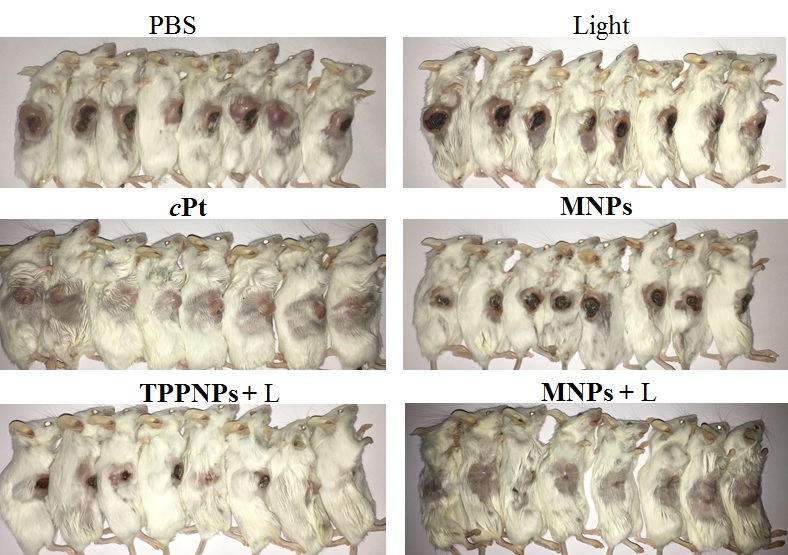


**Supplementary Figure 52.** Photographs of orthotopic 4T1 tumor-bearing mice after treatment with **PBS**, Light, ***c*Pt**, **MNPs**, **TPPNPs** + L, or **MNPs** + L. The irradiation density was 0.3 W cm^–2^ at 671 nm, and the irradiation time was 10 min.


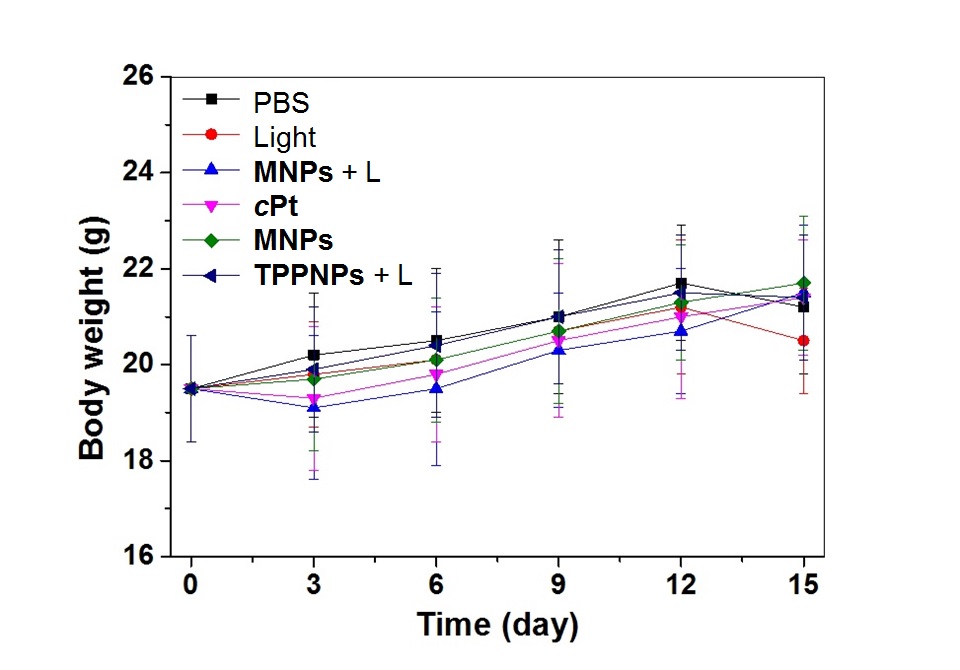


**Supplementary Figure 53.** Body weight changes of orthotopic 4T1 tumor-bearing mice after different formulations. The irradiation density was 0.3 W cm^–2^ at 671 nm, and the irradiation time was 10 min.


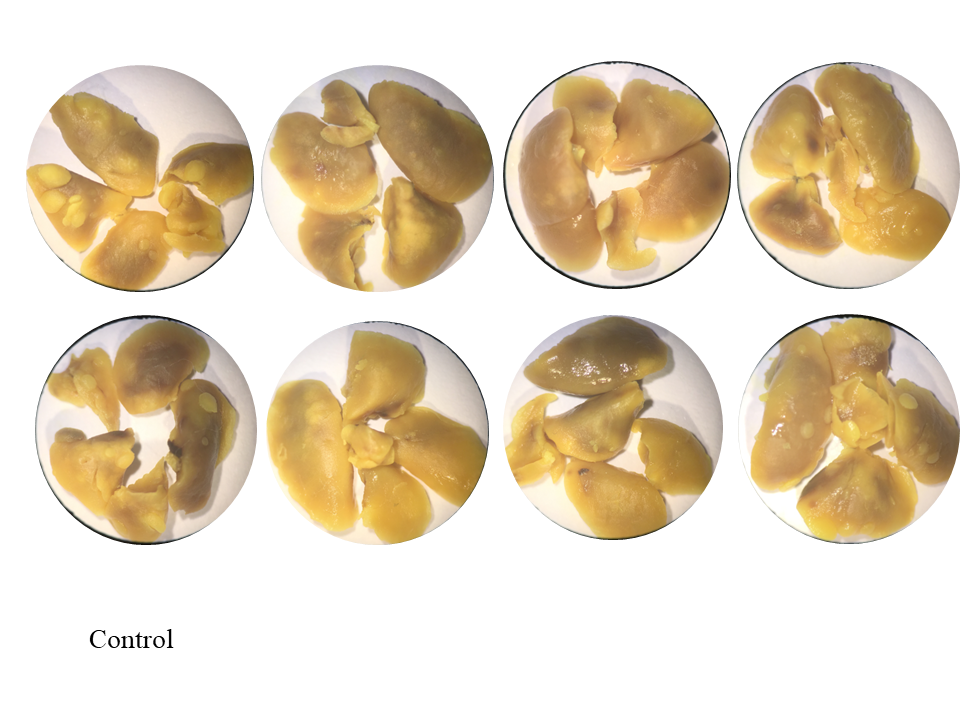


**Supplementary Figure 54.** Photographs of lung tissues from the mice treated with PBS.


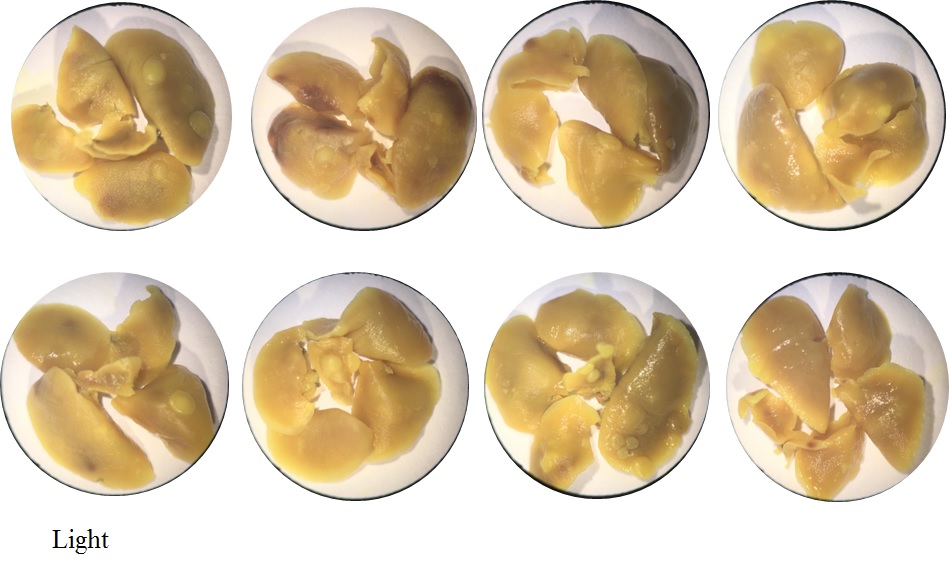


**Supplementary Figure 55.** Photographs of lung tissues from the mice treated with light.


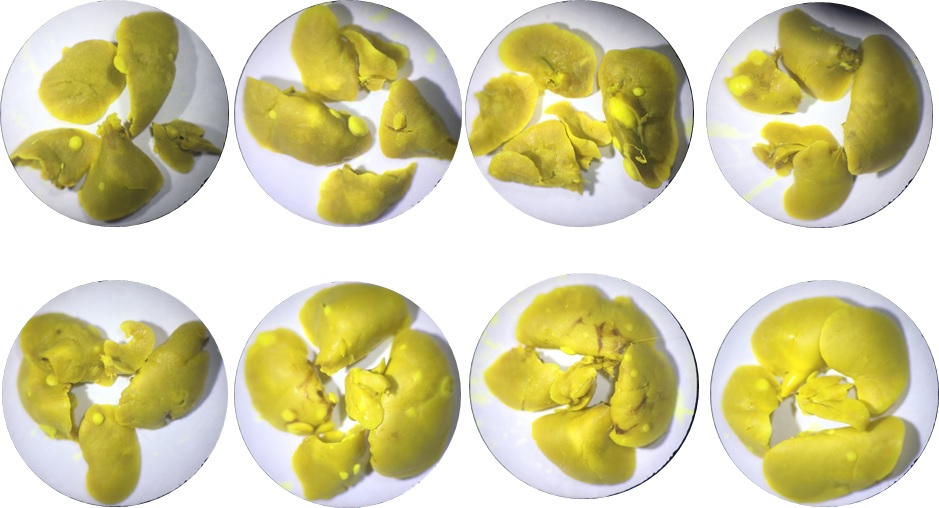


**Supplementary Figure 56.** Photographs of lung tissues from the mice treated with ***c*Pt**.


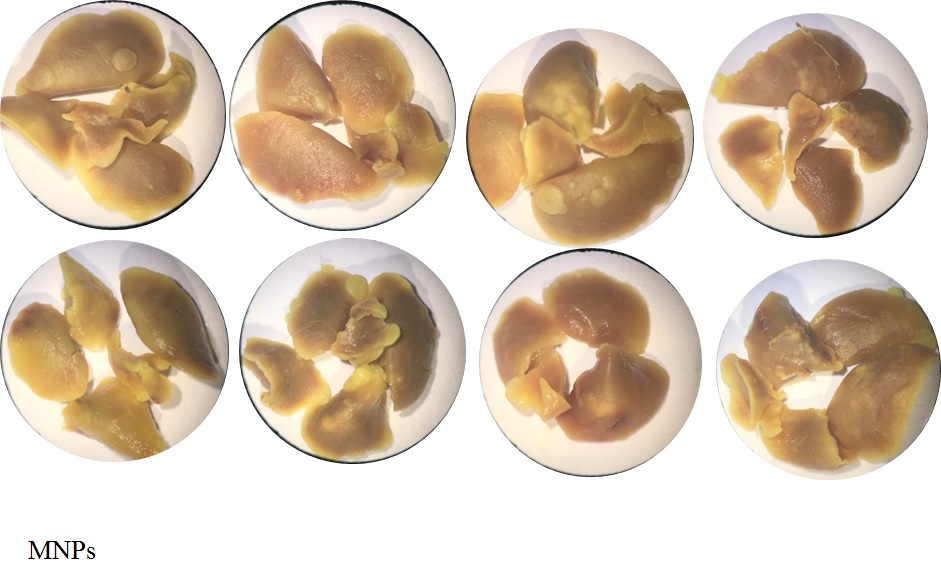


**Supplementary Figure 57.** Photographs of lung tissues from the mice treated with **MNPs**.


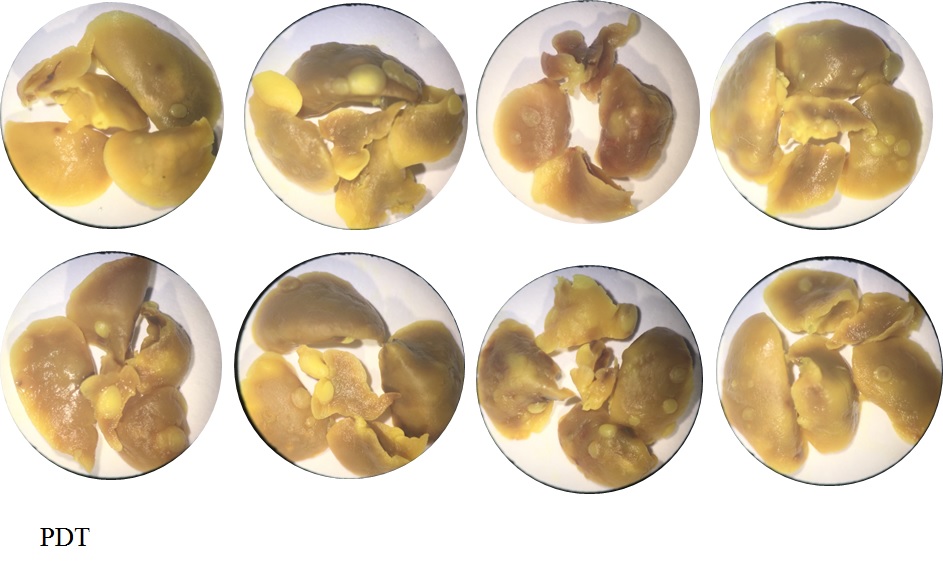


**Supplementary Figure 58.** Photographs of lung tissues from the mice treated with **TPPNPs** + L. The irradiation density was 0.3 W cm^–2^ at 671 nm, and the irradiation time was 10 min.


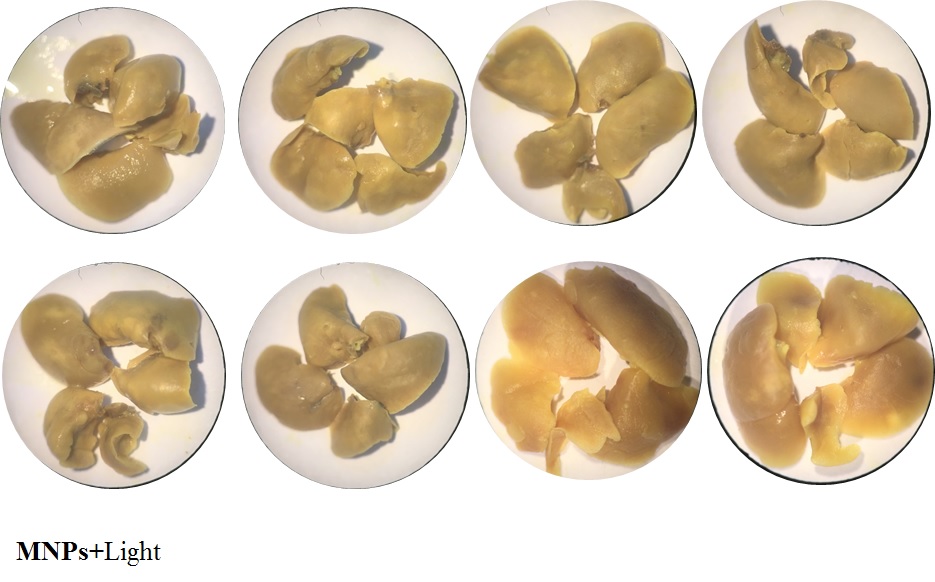


**Supplementary Figure 59.** Photographs of lung tissues from the mice treated with **MNPs** + L. The irradiation density was 0.3 W cm^–2^ at 671 nm, and the irradiation time was 10 min.


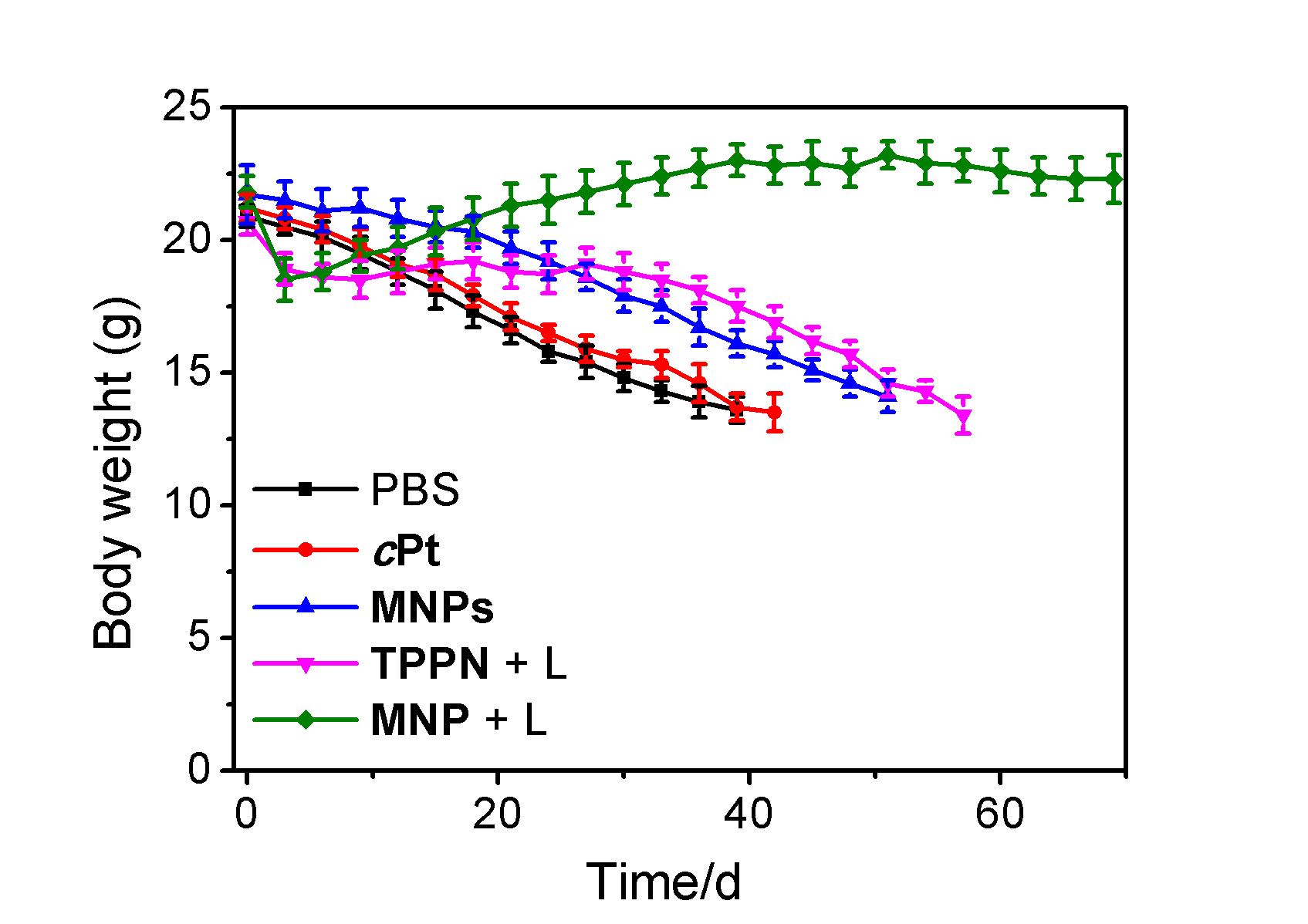


**Supplementary Figure 60.** Body weight changes of LM3 tumor-bearing mice after different formulations. The irradiation density was 0.3 W cm^–2^ at 671 nm, and the irradiation time was 10 min.

*References*

S1 Jude, H. *et al*. [Coordination-driven self-assemblies with a carborane backbone](http://pubs.acs.org/doi/10.1021/ja053050i). *J. Am. Chem. Soc.* **127**, 12131–12139 (2005).

S2 Zhang, F. *et al*. Improving paclitaxel delivery: *In vitro* and *in vivo* characterization of PEGylated polyphosphoester-based nanocarriers. *J. Am. Chem. Soc*. **137**, 2056−2066 (2015).

S3 Cheng, Y. *et al*. Highly efficient drug delivery with gold nanoparticle vectors for in vivo photodynamic therapy of cancer. *J. Am. Chem. Soc*. **130**, 10643–10647 (2008).

S4 Naik, A. *et al*. Visible-light-induced annihilation of tumor cells with platinum-porphyrin conjugates. *Angew. Chem. Int. Ed*. **53**, 6938–6941 (2014).

S5 Yu, M. & Zheng, J. Clearance pathways and tumor targeting of imaging nanoparticles. *ACS Nano* **9**, 6655–6674 (2015).
